# An atomistic mechanism for elasto-plastic bending in molecular crystals†

**DOI:** 10.1039/d2sc06470g

**Published:** 2023-02-13

**Authors:** Biswajit Bhattacharya, Adam A. L. Michalchuk, Dorothee Silbernagl, Nobuhiro Yasuda, Torvid Feiler, Heinz Sturm, Franziska Emmerling

**Affiliations:** a BAM Federal Institute for Materials Research and Testing Richard-Willsätter-Strasse 12489 Berlin Germany biswajit.bhattacharya@bam.de dorothee.silbernagl@bam.de; b School of Chemistry, University of Birmingham Birmingham B15 2TT UK a.a.l.michalchuk@bham.ac.uk; c Japan Synchrotron Radiation Research Institute (JASRI) Hyogo Japan; d Department of Chemistry, Humboldt University Berlin Germany

## Abstract

Mechanically flexible single crystals of molecular materials offer potential for a multitude of new directions in advanced materials design. Before the full potential of such materials can be exploited, insight into their mechanisms of action must be better understood. Such insight can be only obtained through synergistic use of advanced experimentation and simulation. We herein report the first detailed mechanistic study of elasto-plastic flexibility in a molecular solid. An atomistic origin for this mechanical behaviour is proposed through a combination of atomic force microscopy, μ-focus synchrotron X-ray diffraction, Raman spectroscopy, *ab initio* simulation, and computed elastic tensors. Our findings suggest that elastic and plastic bending are intimately linked and result from extensions of the same molecular deformations. The proposed mechanism bridges the gap between contested mechanisms, suggesting its applicability as a general mechanism for elastic and plastic bending in organic molecular crystals.

## Abstract 2

Mechanically flexible single crystals of molecular materials offer potential for exciting new directions in advanced materials design. Unravelling the mechanisms of this fascinating bending phenomenon is needed and developed here.

## Graphical abstract

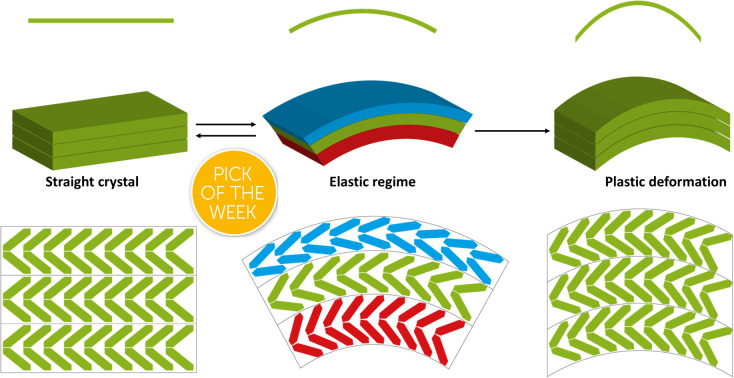

## Introduction

The design of ordered crystalline molecular materials with high mechanical flexibility is a ‘holy grail’ of state-of-the-art materials research.^1,2^ The combination of mechanical flexibility with existing functional properties promises revolutionary advances in next-generation technologies. For example, mechanical actuating technologies,^3,4^ alongside advanced nano-optical^5,6^ and nano-electronic materials,^7^ sensors^8^ and wearable devices^9^ are envisioned. Unfortunately, most crystalline materials are brittle, casting significant barriers over the design of transformative single crystal-based devices.

In recent years, a growing number of crystalline phases have been reported wherein single crystals of small molecules^10–14^ and coordination polymers^15–18^ exhibit a remarkable degree of mechanical flexibility. Both mechanically elastic (reversible)^19^ and plastic (irreversible)^20^ single crystals have been identified, and defined according to the spontaneity of shape recovery upon release of the deforming force. Different mechanically flexible regimes are needed for different applications. Whereas elastically flexible materials are necessary for example as reversible sensors and artificial muscles, plastic flexibility may be useful for shapable nano-devices.^5,21^

Though many mechanically flexible materials have been prepared serendipitously, common structural features have been identified and woven into heuristic crystal engineering strategies.^22^ Elasticity is generally found in crystals which comprise energetically isotropic molecular packing connected by multiple “weak and dispersive interactions”, such as halogen bonding and van der Waals interactions.^1,12^ In contrast, plastic deformation is often observed in crystals with anisotropic crystal packing and low-energy slip planes.^1,20,23–25^ Favourable slip planes have been selectively engineered by introducing weak interactions into the crystal structure *via* the supramolecular “shape synthon strategy”, wherein both the shape and functionality of the molecule are used to engineer crystal packing.^26^ Although powerful, some mechanically flexible materials seem not to adhere strictly to these design strategies.^15,27^ Such findings have prompted growing interest in exploring at the atomic scale how mechanically flexible materials respond to bending,^10,27–29^ and to other related stimuli, such as temperature,^30,31^ anisotropic compression,^32^ and quasi-hydrostatic compression.^17^

Microscopic models for mechanical flexibility have been discussed widely in the literature. Most recently, a mechanism for elasticity has been proposed based on a combination of rotation and displacement of molecules within the lattice.^10,30,31^ For elastic bending, these molecular distortions must remain below some critical threshold. In contrast, models for plasticity in molecular materials typically involve delamination,^28,31^ wherein layers within the crystal glide past one-another to alleviate the stress of bending. It is well known from material mechanics that *all* materials exhibit some degree of elasticity and plasticity prior to fracture. The classification of a material as being *elastically* flexible or *plastically* flexible is therefore synonymous with the experimental resolution of each mechanical regime. This semi-qualitative classification has led to significant debate regarding the mechanical flexibility of some materials, most notably in the case of Cu(acac)_2_.^33^ Elastically flexible materials must either comprise an elastic limit beyond experimental space, or undergo fracture at strains on par with the elastic limit. Similarly, plastically flexible materials are presumably characterised – on average – by remarkably low elastic yield limits.

Designing molecular materials which exhibit experimentally accessible elastic and plastic regimes requires a significant degree of fine-tuning of the crystallographic structure, ensuring a balance between the elastic and rupture yield points. To the best of our knowledge, few molecular materials have been so far reported to exhibit macroscopically detectable elasto-plasticity.^34,35^ A small number of additional examples of elasto-plasticity have been suggested but follow from variations in crystal morphology.^36,37^ Reddy *et al.* noted recently^38^ that understanding this elasto-plastic behaviour holds the key to understanding the structural origin of mechanical flexibility. Although initial studies were performed, detailed mechanistic insight into this mechanical behaviour is still lacking.^39,40^

We herein report the combined experimental and theoretical investigation of macroscopic elasto-plasticity in single crystals of 2,4-dichloro-6-[(6-methylpyridin-2-ylimino)methyl]phenol (DMP). The reported crystal structure of DMP^41^ contains corrugated sheets with halogen–halogen interactions, with simultaneous low-energy slip planes. These structural features represent conventional crystal engineering criteria for both elastic and plastic bending. It follows that DMP is a quintessential model system with which to explore the interplay of these structural features towards designing elasto-plasticity in mechanically flexible molecular materials.

## Results and discussion

**Fig. 1:**
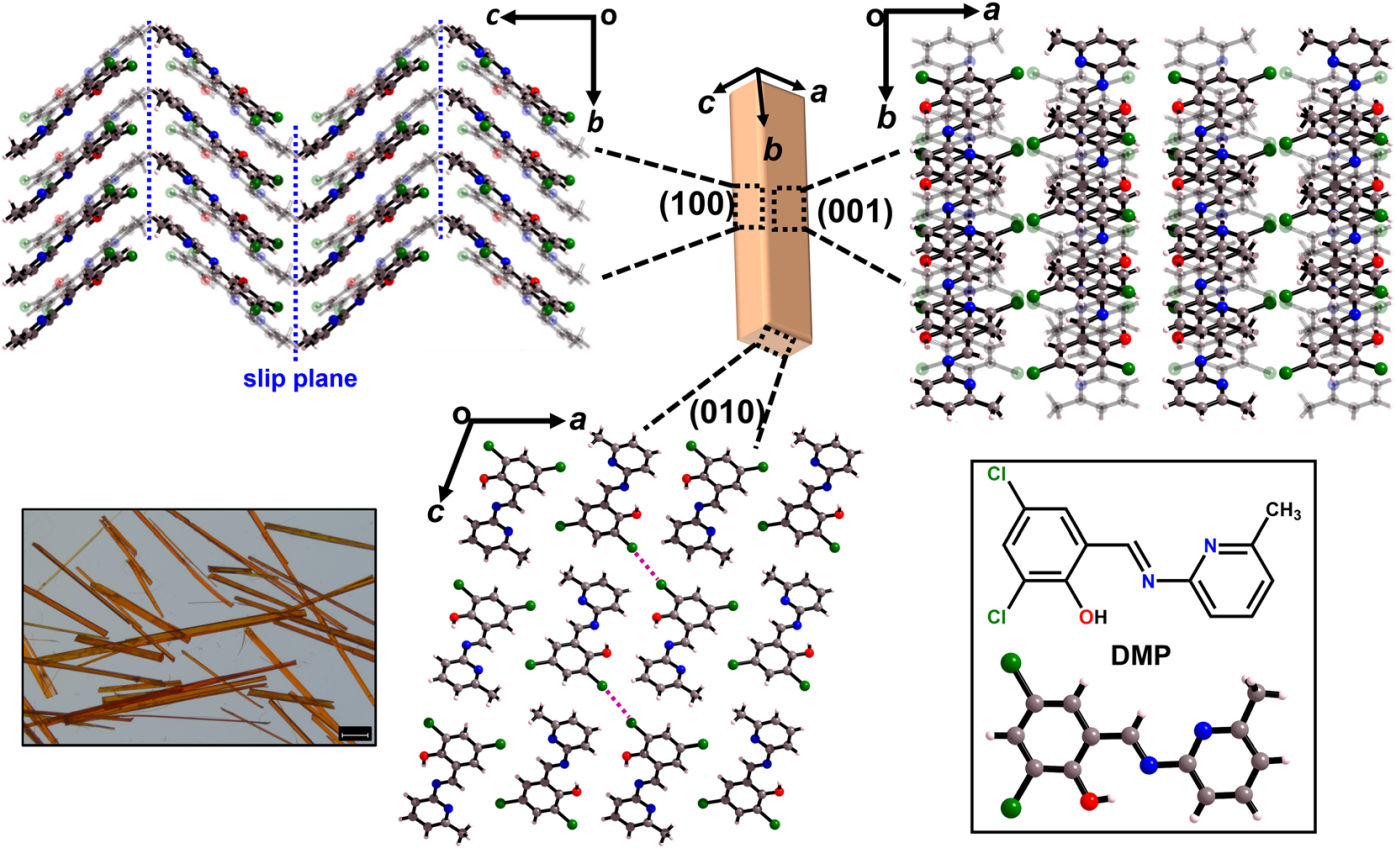
Crystal packing in 2,4-dichloro-6-[(6-methylpyridin-2-ylimino)methyl]phenol (DMP). Crystal morphology with the face indices obtained by single crystal X-ray diffraction. Crystal packing as viewed on the (100), (010), and (001) faces. Potential slip planes and type-I Cl⋯Cl halogen interactions between adjacent herringbone chains (3.39 Å, *θ*_1_ = *θ*_2_ = 161.85°) are shown by blue and magenta dotted lines, respectively. Optical microphotograph of DMP crystals is shown as inset on the left with scale bar 2.5 mm. The structural formula and ball and stick representation of DMP are shown as an inset on the right.

Acicular single crystals of DMP crystallise in monoclinic space group *P*2/*c* with one molecule in the asymmetric unit. The two major faces of DMP crystals are the (100)/(1̄00) and (001)/(1̄00) surfaces, with the minor face being the (010)/(01̄0) surface (Fig. 1 and S3.1†). The molecules crystallise as herringboned chains, which connect *via* type-I Cl⋯Cl halogen bonding interactions to form a corrugated sheet in the *ac*-plane (Fig. 1 and S3.2†). The corrugated sheets are slip stacked along the [010] direction (*i.e.* along the crystal growth axis) *via* π⋯π interactions (Fig. 1). There is a likely slip plane running through the crystal packing. The weak hydrophobic –CH_3_ and –Cl groups which form the herringboned chains provide a slip plane in the *ab*-plane, Fig. 1.

The crystal packing of DMP satisfies the conventional criteria of both elastic and plastic bending. Corrugated crystal packing with weak, dispersive interactions (*e.g.* Cl⋯Cl halogen interactions) often leads to elastic deformation (Fig. 1).^1,12,22^ In contrast, the presence of obvious slip planes is generally considered the principal prerequisite for plastic deformation, Fig. 1.^26^ Hence, from crystal engineering criteria alone it is not immediately apparent what regime of mechanical flexibility to expect from DMP crystals.

**Fig. 2:**
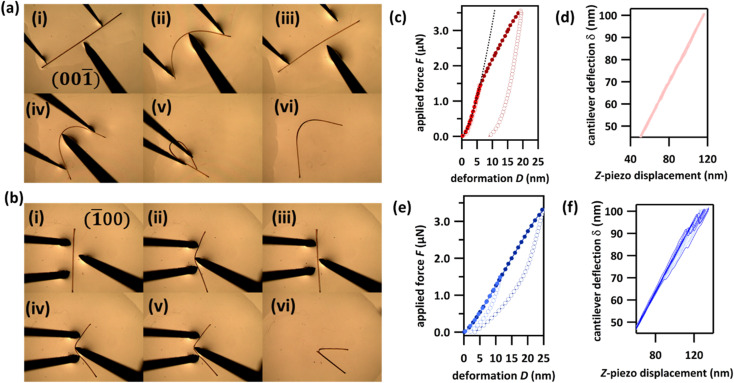
Mechanical properties of DMP single crystals. (a and b) Optical microscope photographs of three-point bending experiments of single crystals of DMP over the (a) (001̄), and (b) (1̄00) crystallographic faces. Note that bending over the (001̄) face is fully reversible below a certain load (i–iii). With increasing load, it exhibits plastic deformation (iv–vi). (c–f) Atomic force microscopy (AFM) force–deformation curves (FDC) for the (c) (001̄) and (e) (1̄00) crystallographic faces, alongside the partial corresponding raw deflection–displacement curves for (d) (001̄) and (f) (1̄00). AFM FDCs were measured using a 150 ± 10 nm radius; the AFM tip was made from electron beam deposited high density carbon. A fit to Hertz theory estimates the Young's modulus of DMP over the (001̄) face (see black dashed line in (c)) to be 5.7 GPa (Poisson ratio *ν* = 0.3). Averaged curves from at least 30 curves are presented. Filled markers in (c) and (e) correspond to the approach part of the measurements, empty circles to the retract part. The experiments with applied maximal forces *F* < 1.5 μN are presented in light red (c) and light blue (e), experiments with applied maximal forces *F* > 1.5 μN are presented in dark red (c) and dark blue (e).

The mechanical flexibility of DMP single crystals was explored by three-point bending experiments in which forceps restrain either end of the crystal while a needle exerts a force between them, Fig. 2. Consistent with the herringboned crystal packing, three-point bending over the (001)/(001̄) face revealed a remarkable degree of elasticity and the crystal could be bent repeatedly into a semi-circle (Fig. 2a, S4.1 and Video S1†). Macroscopically, the crystal returned to its original shape when the deforming force was removed, without visible signs of plasticity. Our measurements suggest typical elastic strain in DMP of *ε* ≈ 2.5% (Fig. S4.3†). The continued bending of DMP crystals into a ‘loop’ was also possible, Fig. 2a(v), without any detectable fracture. However, crystals bent to such a degree darkened in colour, thereby suggesting loss of single crystallinity within the bent region of the ‘looped’ crystals, Fig. 2a(vi). This colour change was accompanied by a significant degree of plasticity (albeit with noticeable residual elasticity), with the crystal remaining bent when the perturbing force was released. The observed plasticity is consistent with the crystallographic slip planes observed in DMP. The behaviour of bending over the (100)/(1̄00) face appeared to be different (Fig. 2b, S4.2 and Video S2†). A small initial regime of elasticity was observed, wherein crystals returned to their nascent state when the deforming load was released. With increased bending, the convex surface began to fracture, whilst the concave surface remained intact and seemingly flexible, Fig. 2b(vi). We note that the qualitative mechanical properties of DMP were retained even at liquid nitrogen temperatures (see ESI for details description in Section S5 and Videos S3–S5†), indicating that DMP may be suitable for applications in flexible thermal-sensors or thermo-optical devices.^42^

We further explored the mechanical behaviour of DMP crystals by measuring force–displacement curves (FDC) *via* atomic force microscopy (AFM), Fig. 2c–f. Our FDC analysis was performed within the framework of Hertz theory, which assumes the interaction between two non-adhesive, continuous and non-conforming surfaces that are deformed within their elastic limits. Hertz theory is widely used for material mechanical analysis where small loads are being applied to areas significantly smaller than the crystal surface, and is therefore optimal for interpreting FDCs measured on flexible crystals. For DMP, deformations for loads < 1.5 μN measured on the crystallographic (001) face are very well described by Hertz theory for elastic deformation (black hashed line in Fig. 2c and S6.1†). This elastic behaviour is supported by the lack of *F*–*D* hysteresis upon removal of the load (d*D*(*F* = 0) < 1 nm), Fig. 2c. Consistent with the macroscopic three-point bending experiments (Fig. 2a), plastic deformation was observed at increased loads > 1.5 μN. This plastic deformation is marked by the typical deviation from Hertz theory and is evidenced further by the significant hysteresis observed during retraction of the applied load (d*D*(*F* = 0) = 9 nm). In contrast, FDCs measured on the (100) face show a different mechanical behaviour. For even low applied forces (<1.5 μN) the deformations cannot be described by Hertz theory and show an initial yield which is confirmed by the *F*–*D* hysteresis (d*D*(*F* = 0) ≈ 1 nm), Fig. 2e. This behaviour is presumably due to surface defects of the crystal. However, for higher forces (>1.5 μN) the FDCs show a zigzag behaviour of the cantilever deflection *δ*, which is typical of sudden, brittle failure (Fig. 2f, curves showing zigzag behaviour are emphasized in dark blue). For comparison curves measured on (001) do not show any zigzag behaviour (Fig. 2c, light red). Yet despite this textbook sign of brittle fracture, significant elasticity is observed upon release of the applied load, as can be seen in the *F*–*D* plot (Fig. 2e). This highly unusual phenomenon is suggestive of the self-healing of micro-fractured DMP crystals and is presumably the origin of the apparent macroscopic elasticity observed during three-point bending over the (100) face (Fig. 2b).^43–45^ Further dedicated studies of this exceptional phenomenon of apparent self-healing are ongoing but suggest that macroscopic elasticity over the two crystallographic faces of DMP may in fact result from distinctly different atomistic origins.

**Fig. 3:**
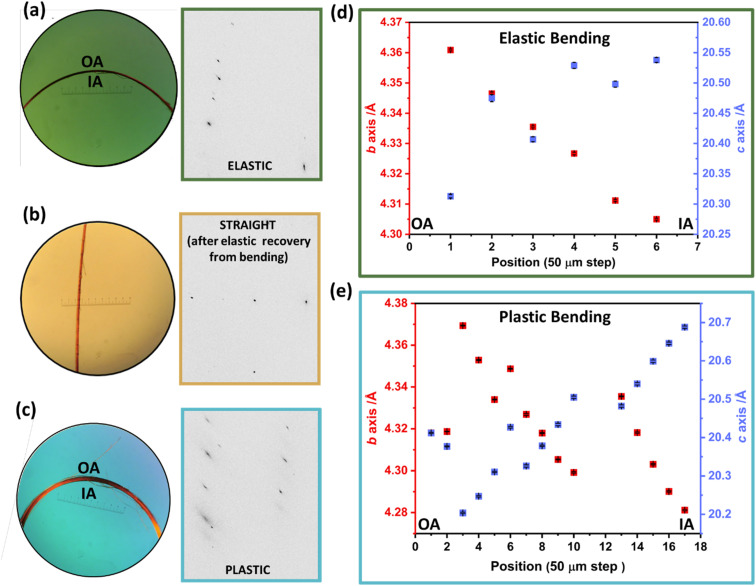
Synchrotron-based μ-focus X-ray diffraction of mechanically bent single crystals of DMP. (a) Photograph of elastically bent single crystal alongside an excerpt of the Bragg scattering obtained from within the bent region of the crystal. (b) Photograph of the elastically bent crystal after release of one end, alongside the associated Bragg scattering obtained from the formerly bent region. (c) Photograph of a thicker second, plastically bent crystal and the associated Bragg scattering. (d) Unit cell parameters derived from diffraction across the elastically bent crystal, from the outer arc (OA) to the inner arc (IA). (e) Unit cell parameters derived from diffraction collected across the plastically bent crystal, from the outer arc (OA) to the inner arc (IA).

We focused our further studies on the unusual elastoplastic behaviour of the (001) face of DMP single crystals. This elastoplastic behaviour occurs without detectable fracture, and its mechanism therefore resides in distortions of the crystal structure itself. Using synchrotron-based μ-focus X-ray diffraction (XRD) with a spot size of 2.76 μm × 0.994 μm we could isolate scattering from localised positions across the bent region of the single crystals, Fig. 3. Two crystals were analysed, one bent to within the elastic regime, and a second bent to within the plastic regime. Scattering obtained from the elastically bent crystal showed noticeable anisotropic broadening of Bragg reflections. This broadening was lost when one end of the crystal was subsequently released and the crystal returned to its straight shape, thereby confirming data were indeed collected within the elastic regime of the crystal, Fig. 3a and b. In contrast, data collected from the plastically bent crystal exhibit marked broadening of the Bragg reflections, which remained following release of the crystal, Fig. 3c.

For the elastically bent crystal, diffraction data were collected from six positions at 50 μm steps across the bent region, Fig. 3a and d. The crystallographic *b*-axis (along the long axis of the crystal) becomes systematically smaller from the outer to the inner arc of the crystal. This is consistent with the proposed deformation of a previously reported elasto-plastic crystal.^38^ Simultaneously, the crystallographic *c*-axis (parallel to the bending vector) elongates over the same region, Fig. 3d. This is consistent with μ-focus XRD studies for elastically bent Cu(acac)_2_ crystals.^10^ No systematic changes were observed in the crystallographic *a*-axis (see ESI Fig. S7.1†). The larger crystal dimensions of the plastically bent crystal allowed for more data points to be collected, Fig. 3c and e. Previous reports of μ-focus XRD measurements on plastically bent crystals, including hexachlorobenzene, suggest minimal changes in unit cell parameters occur during plastic bending.^17,28^ In this light, we were surprised to see such marked and systematic variation in the unit cell response across the plastically bent region, similar to that observed for the elastically bent crystal (Fig. S7.2†). We suspect the observed semi-stochastic trend may be indicative of some residual elasticity of the single crystal, consistent with our macroscopic three-point bending experiments (see Fig. 2a(v and vi)). This residual elasticity, which presumably owes to the herringbone packing of DMP perpendicular to the crystal axis, could explain the difference in unit cell trends in plastically bent DMP as compared with hexachlorobenzene.^20^ These different unit cell responses to plastic deformation may therefore reflect unique local structural origins of plasticity in the two systems. Further dedicated work on the local structure of plastically bent crystals is needed to investigate this phenomenon further.

**Fig. 4:**
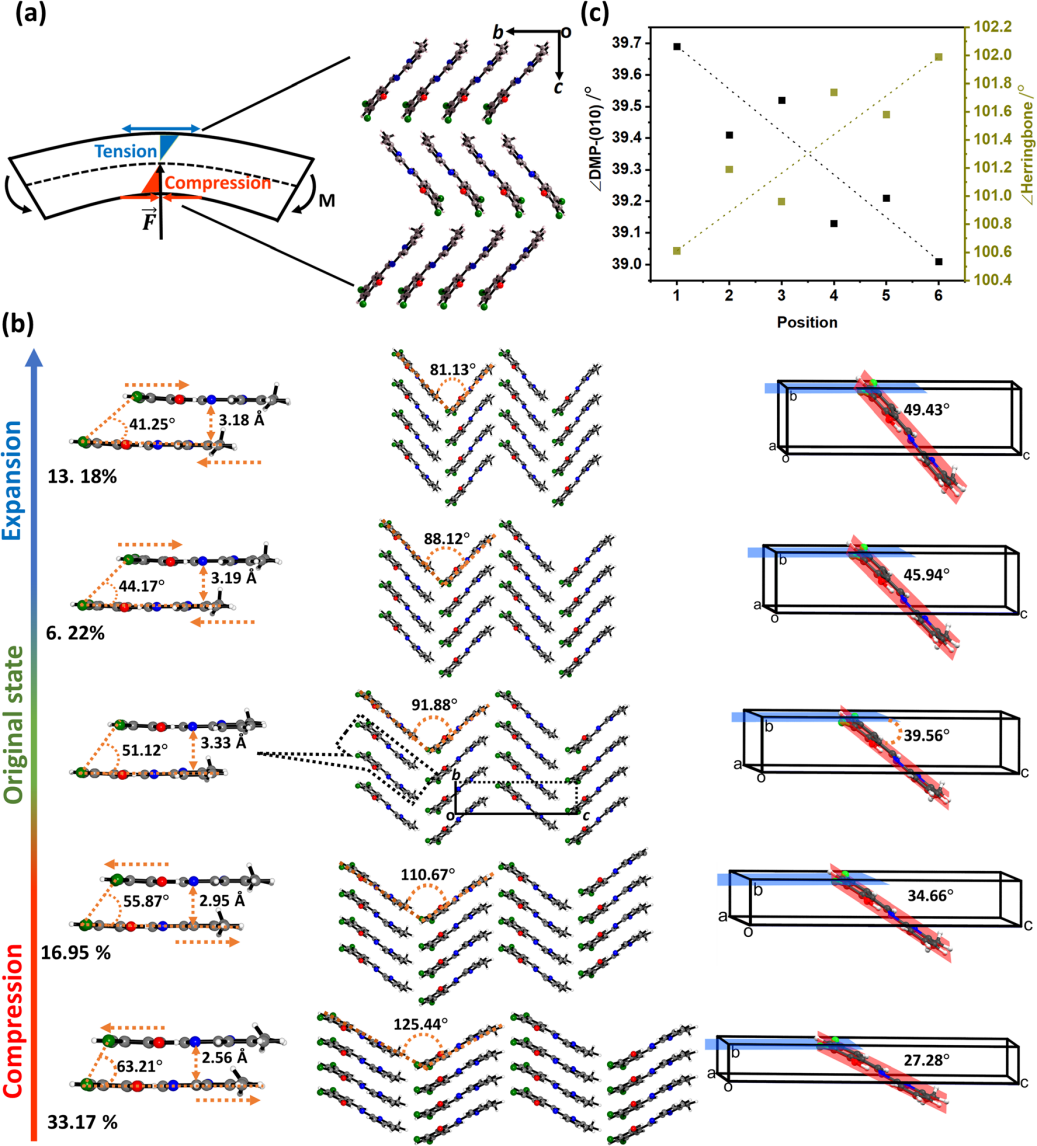
Bending model for elastically flexible single crystals. (a) Ideal bending model for a beam, in which the impinging force, *F⃑*, causes a bending moment, *M*, driving compression at the concave face of the beam and expansion at the convex face of the beam. A neutral axis (dashed line) passes through the centre of the crystal, in which no deformation occurs. (b) The angle between DMP molecules and the (010) plane, and the angle of the herringbone layer, as obtained from experimental μ-focus XRD data. (c) *Ab initio* (PBE-D2) investigation of the anisotropic compression and expansion of DMP single crystals along the crystallographic *b*-axis. We note that extension and compression have been exaggerated to facilitate visualisation of the structural response.

μ-Focus XRD results clearly indicate that the lattice distorts in response to bending. However, μ-focus Raman spectra showed no shift in the vibrational bands across the bent region in either elastically or plastically bent single crystals of DMP (Fig. S8.1†). It follows that no significant structural transformations of the molecules occur during bending. Hence, our combined analyses strongly indicate that bending has an external (lattice), rather than an internal (molecular), origin. Unfortunately, direct evidence for this mechanism is impeded by the high mosaicity of our μ-focus XRD data, which does not allow for robust molecular models for the distorted unit cells (see ESI S7† and Fig. 4). Correspondingly, we turned our attention to *ab initio* simulations to provide atomic level insight into how the DMP molecules adapt to mechanical bending.

The observed systematic variation in unit cell parameters (Fig. 3) is highly reminiscent of the ideal bending of a beam in which there is concerted compression and tension across the bending region of the material, Fig. 4a. Concave deformation accompanies compression perpendicular to the bending moment (*M*), whereas tensile stresses occur over the convex deformation. At the centre of the bend is the so-called *neutral axis*, where no net deformation occurs. Bending of the DMP crystals over either the (100) or (001) face occurs perpendicular to the crystallographic *b*-axis. We therefore expect compression and expansion of this *b*-axis to result.

**Fig. 5:**
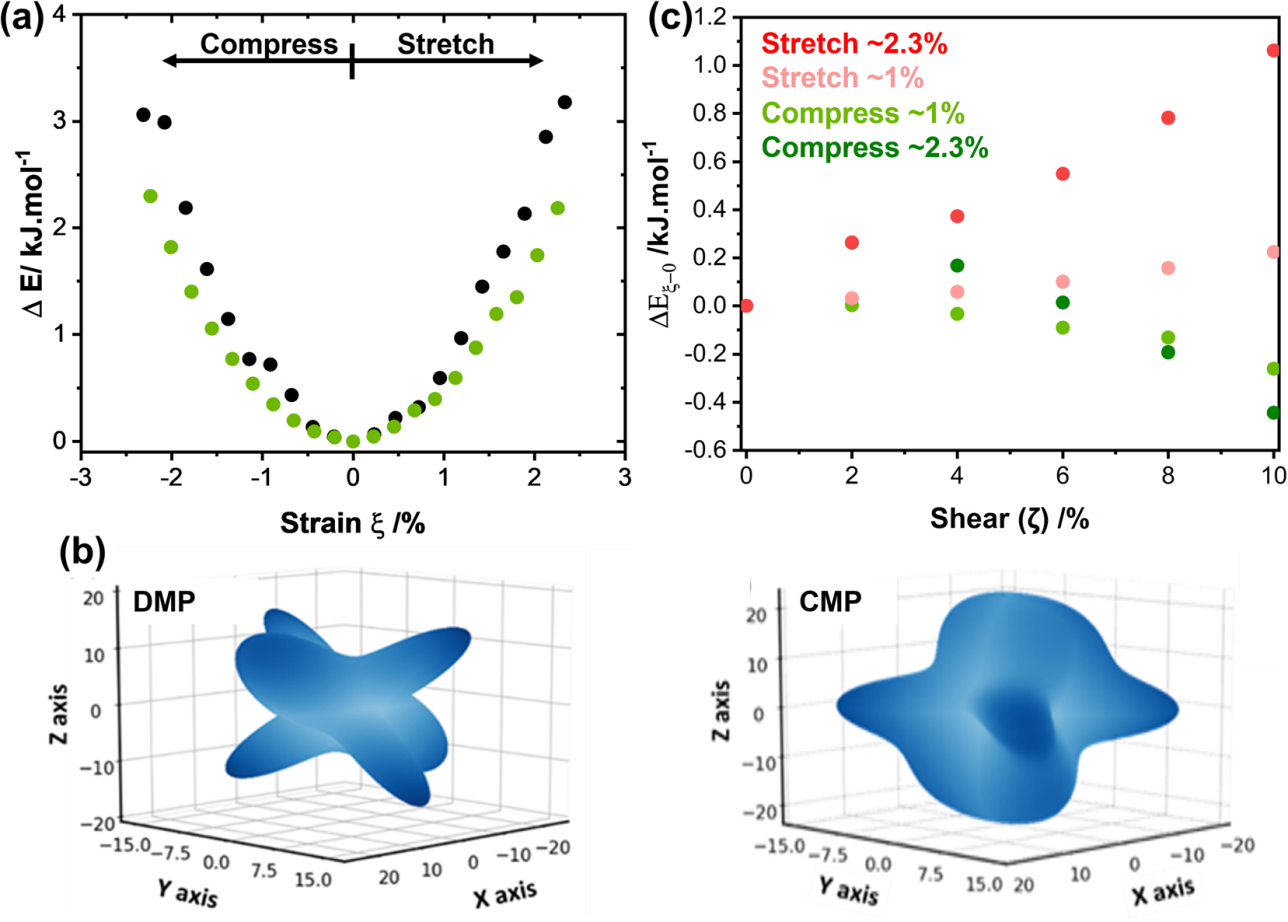
Energetic considerations of the mechanical behaviour of model molecular organic materials. (a) The effect of uniaxial compression (*ξ* < 0) and tension (*ξ* > 0) for elasto-plastic DMP (black) and plastic CMP (green). (b) 3D projection of Young's modulus for (left) DMP and (right) CMP simulated at HF-3c level of theory and visualised using a modified version of ELATE.^48^ Note during bending DMP is compressed along the *b*-axis (Cartesian *y* axis), while CMP compresses along the *a*-axis (Cartesian *x* axis).^47^ Unit cell Cartesian projections for DMP are *a* = (14.541, 0,0), *b* = (0, 4.276, 0), *c* = (−3.80, 0, 19.150); for CMP *a* = (4.376, 0,0), *b* = (0, 18.603, 0), *c* = (−0.543, 0, 13.527). Full elastic tensors are given in ESI S13.† (c) Slip plane (shear plane) energies (PBE-D2) for uniaxially strained (*ξ* in (a)) DMP, relative to the slip plane energy in the unstrained structure, Δ*E*_*ξ*−0_. Absolute slip plane energies are shown in ESI S12.†

We systematically expanded or contracted the cell along the *b*-axis and allowed the remaining geometric parameters and atomic positions to fully relax, Fig. 4b. Anisotropic compression of the unit cell (*i.e.* to simulate the concave crystal face) has the pronounced effect of expanding the structure along the crystallographic *c*-axis, fully consistent with our μ-focus XRD results, Fig. 3d. As the *b*-axis is compressed, our simulations show that the molecules re-adjust to reduce intermolecular steric repulsion and hence reduce the energy of the system. This has the effect of flattening the herringbone layers to increase the planarity of π-stacked chains, and hence expanding the *c*-axis. In contrast, during unit cell expansion (simulating the convex crystal face) void space is energetically disfavoured, and the molecular orientation adapts to maximise intermolecular contacts and hence enthalpic stabilisation. As a result, our simulations suggest that the herringbone angle decreases and the π-stacked molecules slide further apart, leading to contraction of the *c*-axis, Fig. 4b. This is again consistent with our μ-focus XRD results, Fig. 3d. Similar molecular-level rearrangements have been suggested previously as a model for elastically flexible molecular crystals based on analyses of variable temperature crystallographic data,^30,31,46^ and for Cu(acac)_2_ single crystals bent within the elastic regime.^10^ We note that our simulations further suggest that compression and expansion of the *b*-axis are associated with approximately equivalent energy penalties, Fig. 5a and ESI S9.† Moreover, we note that the energy associated with uniaxial compression/expansion is markedly higher for DMP than for our previously reported (and isostructural) plastically flexible crystal, 4-bromo-6-[(6-chloropyridin-2-ylimino)methyl]phenol (CMP).^47^ This is reflected in the corresponding computed elastic properties for each material, Fig. 5b, where the 1D compressibility (Young's modulus) along the distorted vector is *ca*. 8 GPa and *ca.* 6 GPa for DMP and CMP, respectively. We can therefore suggest the ease of compression relates to the observed macroscopic flexibility behaviour, and may be predictable with the aid of computation of material elastic tensors.

The simulated geometric rearrangements yield a thinner and longer unit cell within the concave portion of the bent crystal, with a wider and shorter unit cell on the convex side of the crystal. Thus, while the overall width of the crystal remains largely unchanged in the axial direction, the concave face shortens while the convex face grows. Consistent with the conceptual understanding of mechanical bending and μ-focus XRD results, our atomic-level model obtained from *ab initio* simulation provides unambiguous clarity on the response of molecules to mechanical bending in molecular crystals.

The strained geometry observed during elastic bending is akin to a stressed spring, relaxing readily to its equilibrium state when the perturbing force is removed. While accounting for elasticity, this model does not describe the experimentally observed transition of the elastically bent crystal to the plastic regime. As the splaying of molecules under uniaxial compression is reminiscent of proposed plastic bending mechanisms,^28^ we expect that there should be a link – at least for elasto-plastic crystals – between the structural changes that occur during elastic bending, and the subsequent deformations that occur during plastic deformation. Plasticity in flexible molecular crystals is generally discussed in terms of delamination perpendicular to the applied force.^28^ We observe evidence for this delamination in DMP from optical microscopy images, wherein the colour of the crystal visibly changes during plastic bending, Fig. 2a(iv). For bending of DMP on the (001) face, this mechanism requires delamination of layers in the *ab* crystallographic plane, with slippage along the *b*-axis, Fig. 1c.

Without a definitive understanding as to the location of delamination within the structure, an exact energy barrier to deformation cannot be unambiguously identified. However, qualitative structural analysis suggests that only a single slip plane exists in the *ab* plane of DMP. As proof of concept, we therefore consider the effect of uniaxial compression/expansion (of the *b*-axis) on the energy of the slip plane (*E*_slip_) that runs along the *b*-axis in the *ab* plane, Fig. 1c. To model this slip plane, we impose systematic translation of molecules by a factor *ζ* = *γ*/*b*, where *γ* is the Cartesian value of translation across unit cell axis *b*. A value of *ζ* = 1 denotes translation of the full unit cell axis. In its non-stressed state, slip in DMP is associated with an energy barrier of *E*_slip_ ≈ 2.0 kJ mol^−1^ for *ζ* = 5%, increasing to *E*_slip_ ≈ 7.5 kJ mol^−1^ for *ζ* = 10%. These are reasonably small energies on the order of thermal energy (RT ≈ 2 kJ mol^−1^), though we note that such small slip plane energies are only achieved where the molecules are allowed to rotate in response to the applied strain. This suggests that the slip plane is not a true slip plane, but rather represents a ‘hindered’ slip plane in the structure.

Our simulations suggest that uniaxial mechanical strain, of a similar magnitude to that observed upon bending (see Fig. 3), is enough to modify *E*_slip_ of DMP. Representing the outer arc of the crystal, expansion of the *b*-axis (*ξ* ≈ 1%, Fig. 5a) causes *E*_slip_ to increase by ∼0.25 kJ mol^−1^ for a shear distortion of *ζ* = 10%. The slip plane energy increases further, Δ*E*_slip_ ≈ 1 kJ mol^−1^, for the same shear distortion where larger expansions are considered (*ξ* ≈ 2.3%), Fig. 5c. Thus, our results suggest that expansion (tension) of the unit cell on the outer arc disfavours plastic deformation *via* slippage. In contrast, compression of the *b*-axis appears to favour slip. Comparing energies required for 10% shear (*ζ* = 0.1), compression of the *b*-axis by *ξ* ≈ 1% reduces slip energy by Δ*E*_slip_ ≈ −0.2 kJ mol^−1^, with larger compressions (*ξ* ≈ 2.3%) decreasing the slip energy even further, Δ*E*_slip_ ≈ −0.5 kJ mol^−1^.

**Fig. 6:**
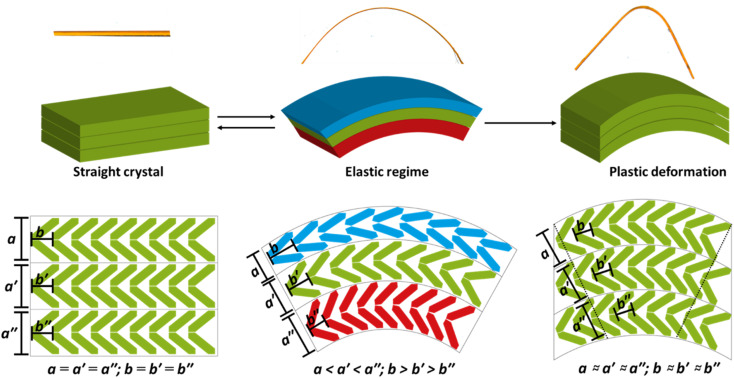
Schematic mechanisms for mechanical flexibility in DMP. Elastic bending regime. As the crystal bends, a region of high pressure (red) is generated at the concave face, with a region of low pressure (blue) generated at the convex face. The former leads to shortening of the crystal along the bending axis (010), and elongation along the perpendicular axis. Simultaneously, the low-pressure region at the convex face leads to growth of the (010) axis, and shortening of the other crystal direction. Plastic bending regime. Once the accumulated strain overcomes the slip plane energy (shown as curved horizontal grey lines), delamination along this slip plane occurs until balance between compression and slip plane energies is restored (hence residual elasticity in plastic regime).

There is an intimate link between the elastic and plastic bending nature in flexible crystals, and in fact that both behaviours may be linked through the same molecular origins. While the effect is small in our highly idealised model, it appears that anisotropic compression that occurs during the initial stages of bending causes molecules to rearrange. Where packing is favourable, this rearrangement can reduce energy barriers associated with subsequent, larger deformations like delamination. Hence, the mechanically driven distortions that occur during elastic bending create the conditions for subsequent plastic deformation, Fig. 6. Considering multi-dimensional deformations in this way not only demonstrates a direct link between elastic and plastic deformations in molecular crystals, but indicates that both phenomena are intimately linked and based on extensions of the same molecular-level deformations. That said, while a step-wise deformation-delamination mechanism may be suitable to describe elasto-plastic transitions in this class of crystal, strain is unlikely to accumulate in ‘plastically flexible’ crystals to cause initial uniaxial strain. Instead, we suspect that plastically flexible crystals inherently contain low energy slip planes and bypass the need for significant initial uniaxial distortions.

We highlight that our model, Fig. 6, is consistent with those which discuss crystal elasticity in terms of non-covalent interaction isotropy.^1,12^ Energy framework calculations, ESI S11,† suggest strong pairwise interactions along the π⋯π interactions, with weaker interactions in the orthogonal directions. Our findings strongly suggest that anisotropic energy frameworks can still exhibit marked elasticity, so long as the crystals are not bent beyond the elastic limit. We stress that our model offers a unifying re-interpretation of previous models from the perspective of molecular geometry. In fact, this highlights that model which discuss NCI isotropy and molecular rotation are largely synonymous.

## Conclusions

All materials are characterised by both elastic and plastic mechanical regimes. The classification of a material as being *elastically* or *plastically* flexible is simply a matter of which regime is macroscopically most visible.

We report here a rare example of a mechanically flexible *elasto-plastic* organic molecular crystal, 2,4-dichloro-6-[(6-methylpyridin-2-ylimino)methyl]phenol (DMP). Three point bending of DMP single crystals reveals first a marked elastic regime in which the crystals return to their linear form after release of the stress, with elastic strain *ε* ≈ 2.5%. If bent beyond this limit, the crystal exhibits unmistakable plasticity. Our μ-focus Raman spectra and μ-focus XRD data indicate that bending of DMP single crystals occurs through *external* (lattice) rather than *internal* (molecular) deformation. Consistent with continuum mechanics models of bending, the bending axis (here, the *b*-axis) compresses at the concave face of the crystal and expands at the convex surface. To maximise the enthalpic stabilisation of the material under these deformed conditions, the molecules undergo minor rotations and translations. The internal energy of the crystal increases quickly with compression/tension, and eventually surmounts slip plane energies. At this point, strain energy is released through delamination, leading to the observed plastic regime. Hence, our findings suggest the underlying atomistic origins of elastic and plastic deformation are intimately connected and result from extensions of the same deformations, at least for the case of elasto-plastic crystals.

We expect our model holds for the general case of elastically flexible crystals that comprise herringboned layers which pack perpendicular to the long axis of the crystal. We also expect this model, in general, to apply to other elasto-plastic crystals like Cu(acac)_2_ for which uniaxial compression/tension during elastic bending has been reported prior to the onset of plasticity. Tuning the intra-layer interactions through specific non-covalent interactions will facilitate powerful design strategies for elasto-plasticity in molecular solids. Only with these design strategies can we realise the transformative potential of next generation flexible devices based on molecular materials.

## Data availability

All experimental and simulation data and detailed procedures are available in ESI.†

## Author contributions

B. B., A. A. L. M., and D. S. conceived the project. B. B., and T. F. synthesised the material and conducted the qualitative mechanical bending experiments. D. S. conducted AFM experiments. N. Y. conducted microfocus XRD experiments, and data were analysed by A. A. L. M., and B. B. A. A. L. M., and B. B. conducted the Raman spectroscopy experiments. A. A. L. M. conducted the DFT calculations. B. B., A. A. L. M., D. S., N. Y., T. F., H. S., F. E. discussed the results and commented on the manuscript.

## Conflicts of interest

There are no conflicts to declare.

## Supplementary Material

SC-014-D2SC06470G-s001

SC-014-D2SC06470G-s002

SC-014-D2SC06470G-s003

SC-014-D2SC06470G-s004

SC-014-D2SC06470G-s005

SC-014-D2SC06470G-s006

SC-014-D2SC06470G-s007
