# Supplementary material for: An atomistic mechanism for elasto-plastic bending in molecular crystals
Source: Chem Sci. 2023 Feb 13;14(13):3441–50. doi: 10.1039/d2sc06470g (PMC10056064; doi:10.1039/d2sc06470g)
Supplement: SC-014-D2SC06470G-s001 [file SC-014-D2SC06470G-s001.pdf]

Electronic Supplementary Material (ESI) for Chemical Science.
This journal is © The Royal Society of Chemistry 2023

Electronic Supporting Information

An atomistic mechanism for elasto-plastic bending in molecular crystals

Biswajit Bhattacharya*,†,1, Adam A. L. Michalchuk*,†,1,2, Dorothee Silbernagl*,1, Nobuhiro Yasuda,3 Torvid Feiler,1,4 Heinz Sturm,1 and Franziska Emmerling1,4

1. BAM Federal Institute for Materials Research and Testing, Richard-Willsätter-Strasse 12489 Berlin, Germany

2. School of Chemistry, University of Birmingham, Birmingham B15 2TT, United Kingdom

3. Japan Synchrotron Radiation Research Institute (JASRI), Hyogo, Japan

4. Department of Chemistry, Humboldt University, Berlin, Germany

† These authors contributed equally

Email: biswajit.bhattacharya@bam.de (BB); a.a.l.michalchuk@bham.ac.uk (AM); dorothee.silbernagl@bam.de (DS)

CONTENTS

S1. Experimental Details……………………………………………………………………2

S2. Computational Details…………………………………………………………………..4

S3. Crystallographic Structure of DMP …………………………………………………5

S4. Mechanical Flexibility of DMP ……………………………………………………..9

S5. Thermochromic Behaviour of DMP …………………………………………………11

S6. Force-Displacement Curves – Atomic Force Microscopy ……………………………13

S7. Synchrotron Based Microfocus X-Ray Diffraction ……………………………….. 14

S8. Vibrational Spectroscopy of DMP ……………………………………………………16

S9. Anisotropic Compression of DMP ……………………………………………………19

S10 Crystal Structure of CMP……………………………………………………………...21

S11 Anisotropic Compression of CMP …………………………………………………24

S12. Slip Plane Energies of DMP………………………………………………………..27

S13 Simulated Elastic Tensors…………………………………………………………..32

S13. References……………………………………………………………………………34

S1 EXPERIMENTAL DETAILS

S1.1 Materials| 2-amino-6-methyl pyridine (98%; CAS No:1824-81-3) and 3.5-dichlorosalisaldehyde (98%; CAS No: 90-60-8) were purchased from JK Chemicals and abcr respectively and used as received.

S1.2 Crystal growth| The compound, 2,4-dichloro-6-[(6-methylpyridin-2-ylimino)methyl]phenol (DMP) was synthesized by liquid assisted mechanochemical grinding of equimolecular mixture of 2-amino-6-methyl pyridine (108 mg, 1 mmol) and 3.5-dichlorosalisaldehyde (175 mg, 1 mmol) in a mortar and pestle using 100 μL of methanol. Slow evaporation of a saturated CH_2Cl_2 solution of ground powder at room temperature in beaker yielded long needle-shaped (size: 5 cm x 1 mm x 2 mm) orange coloured crystals.

S1.3 Single Crystal X-ray Diffraction| Single crystals of 2,4-dichloro-6-[(6-methylpyridin-2-ylimino)methyl]phenol were individually mounted on a glass tip. Single crystal XRD data were, collected on a Bruker D8 Venture system with graphite-monochromatized Mo Kα radiation (λ = 0.71073 Å) at 300 K and 100 K. Data reduction was performed with Bruker AXS SAINT[1] and SADABS[2] packages. The structure was solved by SHELXS 2018[3] using direct method and followed by successive Fourier and difference Fourier synthesis. Full matrix least-squares refinements were performed on F^2 using SHELXL-2018[3] with anisotropic displacement parameters for all non-hydrogen atoms. All other calculations were carried out using SHELXS 2018,[3] SHELXL 2018,[3] and WinGX (Ver-1.80),[4] whereas Mercury v3.6[5] was used to visualize and to draw some of the figures for the structures. Detailed data collection strategy and structure refinement parameters along with crystallographic data are presented in **Table S3.1**.

S1.4 μ-Focus Raman spectroscopy| All Raman spectra were collected using a Horiba Jobin Yvon Labram HR800 Raman microscopy system with an Olympus BX41 microscope. A continuous-wave diode-pumped solid-state laser ($\lambda = 633$ nm) was used for Raman excitation. The laser spot was focused onto the sample by a 50×/N.A. = 0.75 objective (N.A. = numerical aperture). The scattered light was collected by the same lens, with the reflected and Rayleigh scattered light filtered by a bandpass filter. The Stokes-shifted Raman-scattered light was dispersed by an 1800 mm^{-1} grating. The signal was detected by a Peltier-cooled (-60 ^oC) charge-coupled device (CCD) camera. The spectrometer entrance slit was 100 μm wide with a confocal pinhole 1000 μm in diameter. With this configuration, the spectral resolution varies from 0.5 cm^{-1} per CCD pixel (at 100 cm^{-1} Raman shift) to 0.3 cm^{-1} per CCD pixel (at 3800 cm^{-1} Raman shift). The laser spot resolution is approx. 1 μm x 20 μm.[1]

S1.5 Atomic Force Microscopy| Atomic force microscopy (AFM) measurements were performed with an MFP-3D Asylum (Oxford Instruments Asylum Research Inc., Santa Barbara, CA). AFM Force distance curves (FDC) were recorded with a frequency of 1 Hz in a force volume with an area of 10 x 10 μm and 10 x 10 measuring points yielding 100 single curves. All FDCs were corrected for the point of contact and subsequently averaged with SOFA. As AFM probe a biosphere B150-NCH (Nanotools GmbH, Munich, Germany) was used with a spring constant kc = 36.5 N/m, determined by a non-invasive thermal noise method. The tip is made from electron beam deposited high density carbon, with a tip radius R = 150nm ± 10nm, a Young's modulus E_{tip} = 350 GPa and a Poisson ratio ν_{tip} = 0.3.

S1.6 Microfocus Synchrotron X-ray Diffraction| Microfocus synchrotron X-ray diffraction data were collected on the precision diffractometer equipped with a hybrid photon counting detector, EIGER X 1M detector (DECTRIS) in the SPring-8 BL40XU beamline. The X-ray beam (λ = 0.81042 Å) was focused to 0.922 (vertical) x 3.67 (horizontal) μm using a zone plate. To irradiate only a focused beam, a 30-μm

diameter sorting aperture and a 40-μm-diameter centre stop were used. One end of the crystal was first glued on a pin and the crystal was bent to form a loop, attaching the second end to the pin by glue. This pin was mounted on the goniometer head for data collection. The beam was focussed on the middle of the bent position. After measurement of diffraction from the centre of the elastically bent crystal, one end of the crystal is released to confirm reversibility of the bending phenomenon. The same diffraction measurements were carried out on the re-straightened crystal, confirming that the crystals returned to their original configuration after removal of the deforming stress. The focused X-ray was incident normal to the bending plane ($\omega = 0°$). The ω range, oscillation angle ($\Delta\omega$) and exposure time were ±10°, 0.1° and 0.5 s, respectively. Data were collected at room temperature. The measured position was moved from the convex to the concave side of the bend in steps of 50 μm, and 500 diffraction images were collected from each position.

S2 COMPUTATIONAL DETAILS

S2.1 Geometry optimisation and phonon simulations| Solid state simulations were performed within the framework of plane wave Density Functional Theory (DFT), as implemented in CASTEP v19.11.[2] Input structures were taken from experimentally determined geometries (See Section S3, S11, and S14). The electronic structure was expanded in plane waves to a kinetic energy cutoff of 1000 eV, and sampled on a Monkhorst-Pack[3] grid of spacing 0.05 $Å^{-1}$. Simulations were performed using the exchange-correlation functional of Perdew-Burke-Ernzerhof (PBE),[4] along with the D2 dispersion correction.[5] Norm-conserving pseudopotentials were generated 'on the fly' as implemented in CASTEP. Convergence on the geometry optimization was accepted with energy convergence < 3 x 10^{-8} eV/atom, electronic eigenvalue convergence < 1 x 10^{-12} eV, residual atomic forces < 1 x 10^{-3} eV/Å, and maximum stress of 1 x 10^{-2} GPa. The charge density was integrated over a fine grid of G_{max}=64.8 A^{-1}, to facilitate convergence of vibrational frequencies. The vibrational frequencies were calculated within the framework of density functional perturbation theory (DFPT) at the Brillouin zone centre ($\boldsymbol{k}$=Γ).[6] LO-TO splitting was not explicitly considered.

S2.2 Elastic Tensors| Elastic tensors were calculated at PBE-D3/def2-mSVP level of theory as implemented in CRYSTAL17.[7] The D3 correction[8] was applied with BJ damping. Following recent indications that computational elastic tensors can be qualitatively produced using semi-empirical methods, we additional calculated the elastic tensors using the HF3c[9] and sHF-3c methods, as implemented in the same software suite. In all cases, the electronic structure was sampled using SHRINK factors 4 4, with convergence of energy gradients of 4E-5 eV. The remaining parameters were held at the default values.

S2.4 Energy Framework Calculations| The energy frameworks calculations[10] relating to intermolecular interactions of DMP were performed using the software suite Crystal-Explorer[11] based on B3LYP/DGDZVP molecular wavefunctions calculated using the CIF files. For calculations, the hydrogen atoms were normalized to standard neutron diffraction values. The energy frameworks constructed were based on the crystal symmetry and total interaction energy components of which included electrostatic, polarization, dispersion and exchange repulsion components scaled by 1.057, 0.740, 0.871 and 0.618, respectively. The interaction energies below 5 kJ.mol^{-1} are omitted for clarity and the cylinder thickness is proportional to the intermolecular interaction energies along the parallel vector passing through the cylinder.

S3 Crystallographic Structure of DMP

S3.1 Face indexing of DMP single crystals| To correlate the macroscopic bending against molecular packing, the orientation of DMP single crystals were indexed by means of single crystal X-ray diffraction, **Fig. S3.1**. Our indexing suggests the three principal faces exist: the major face corresponds to the $(00\bar{1})$ surface, with the minor faces corresponding to the $(\bar{1}00)$ and (010) surfaces.

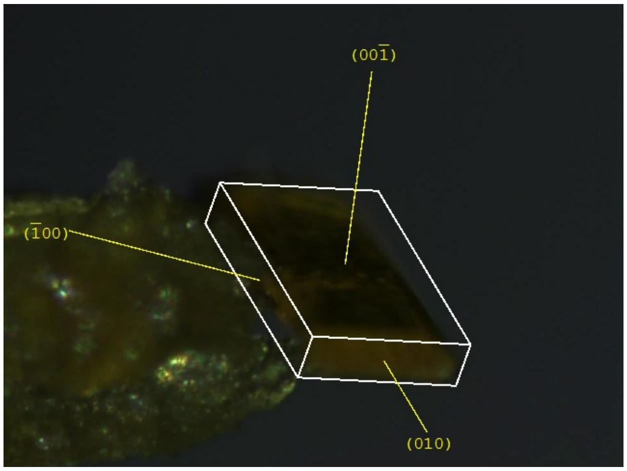

Fig. S3.1. Face indexed image of pristine crystal of DMP.

S3.2 Crystal structure| Single crystal X-ray diffraction experiments were performed at both 300 K and 100 K, **Table S3.1**. The crystal structure packs in herringboned layers running along the crystallographic *c* axis, and π-stacked along the *b*-axis, **Fig. S3.2.**

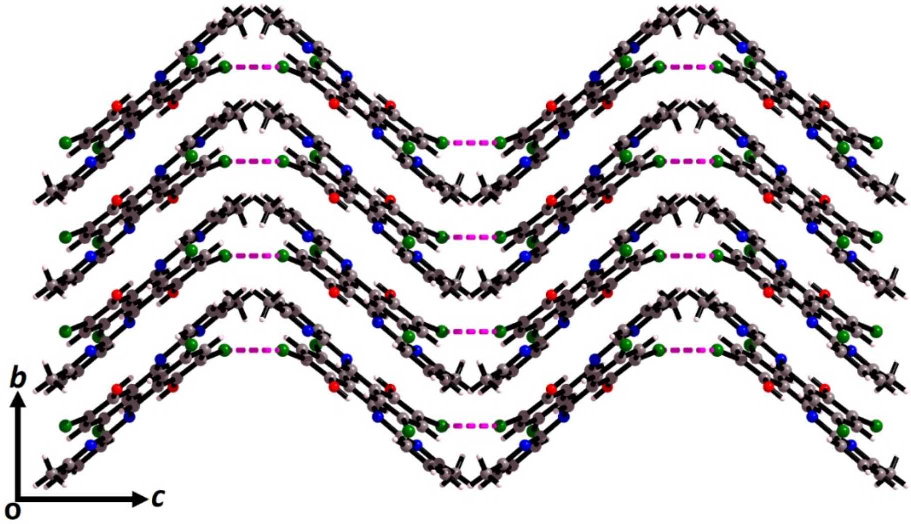

Fig. S3.2. Cl···Cl halogen bonding interactions (cyan dotted lines) in molecular packing diagram of DMP in the (010) plane.

The experimentally obtained unit cell of DMP (see **Table S3.1**) was fully relaxed at PBE-D2 level of theory (see **Section S2**). The relaxed unit cell dimensions are in good agreement with the experimentally obtained values, **Table S3.2**, with the unit cell volume underestimating the 100 K crystal structure by < 3 %. Variable temperature experimental data suggest significant thermal expansion of DMP single crystals, with the 300 K and 100 K structures differing by *ca.* 3%. Hence, this slight underestimation between the DFT (0 K) and experimental (100 K) geometries is expected and reasonable.

Table S3.1. Crystallographic and structural refinement parameters of DMP crystals at 300 K and 100 K

Temperature (K)	300 K	100 K
Formula	$C_{13}H_{10}Cl_2N_2O$	$C_{13}H_{10}Cl_2N_2O$
Formula Weight	281.13	281.13
Crystal System	monoclinic	monoclinic
Space group	P2/c	P2/c
a/ Å	14.5261(17)	14.3805(11)
b/Å	4.3104(5)	4.2807(3)
c/Å	20.361(2)	20.0178(16)
α/°	90	90
β/°	102.088(5)	101.319(3)
γ/°	90	90
V/ $Å^3$	1246.6(3)	1208.30(16)
Z	4	4
D_c/ g cm^{-3}	1.498	1.545
μ /mm^{-1}	0.508	0.524
F(000)	576	576
2θ range/°	2.87-53.51	2.89-55.06
Reflections collected	13090	13587
Independent reflections	2668	2781
R_{int}	0.0369	0.1562
Goodness of fit (F^2)	1.020	1.112
$R1$ ($I > 2σ$(I))	0.0392	0.0512
$wR2$($I > 2σ$(I))	0.1197	0.1339
CCDC No.	2131405	2131406

Table 3.2| Comparison of PBE-D2 optimised geometries against experimental parameters.

	**a**	**b**	**c**	α	β	γ	**V**
Exp 300 K	14.5261	4.3104	20.361	90	102.088	90	1246.6
Exp 100 K	14.3805	4.2807	20.0178	90	101.319	90	1208.30
PBE-D2	14.242	4.298	19.464	90	99.671	90	1174.610
ΔPBE-D2	-0.96%	-0.40%	-2.77%	-	-1.63%	-	-2.79%

The single crystal structures suggest that no major structural changes occur upon cooling to 100 K, **Table S3.1** and **Fig. S3.3**, beyond the thermochromic transition (see **Section S5**). Residual electron density maps at 300 K and 100 K display an obvious change of electron density volume of the enol hydrogen (-O1-H1) with decreasing temperature **(Fig. S3.4**). Residual density suggests proton transfer towards the imine

nitrogen (N1) was not observed. This is likely due to strong scattering of Cl atoms which precludes the detections of presumably partially transfer proton.

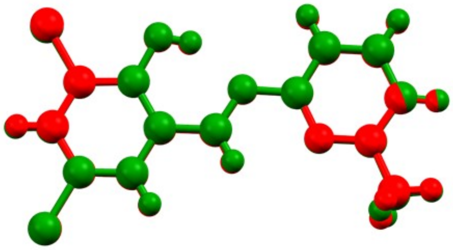

Fig. S3.3. Overlay diagram of DMP molecules in asymmetric unit at different temperature from 100 K (green) and 300 K (red).

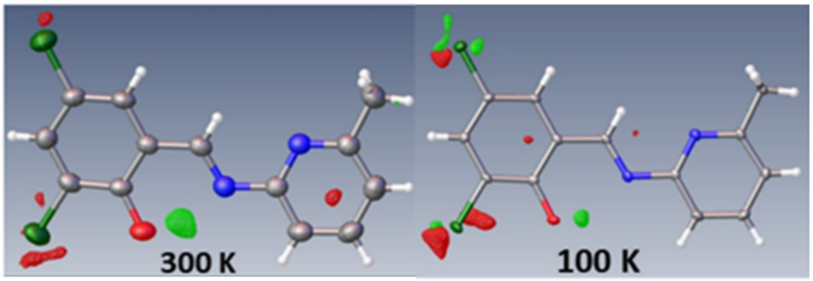

Fig. S3.4. Residual electron density map at different temperature of DMP crystal showing the change of electron density volume of enol hydrogen.

S3.3 Energy Framework Calculations of DMP| To understand the mechanical flexibility of DMP crystals, pairwise total intermolecular interaction energies (E_{total}) were calculated by energy framework calculations, **Fig. S3.5** and **Table S3.3**. The interaction energies due to π–π stacking interactions along the b-axis are the most dominating interactions (−52.5 kJ.mol^{-1}) that stabilizes molecular columns running along the needle axis. In contrast, the interaction energies along the herringbone chain are −21.9 kJ.mol^{-1}, and the interchain interaction (along the a axis) energies are −82.3 kJ.mol^{-1}. Based on the previous model, calculation suggests plasticity should dominate this system. Despite the experimental finding shows marked elasticity before goes to the plastic deformation. This could be attributed due to the zig-zag interlock crystal packing.

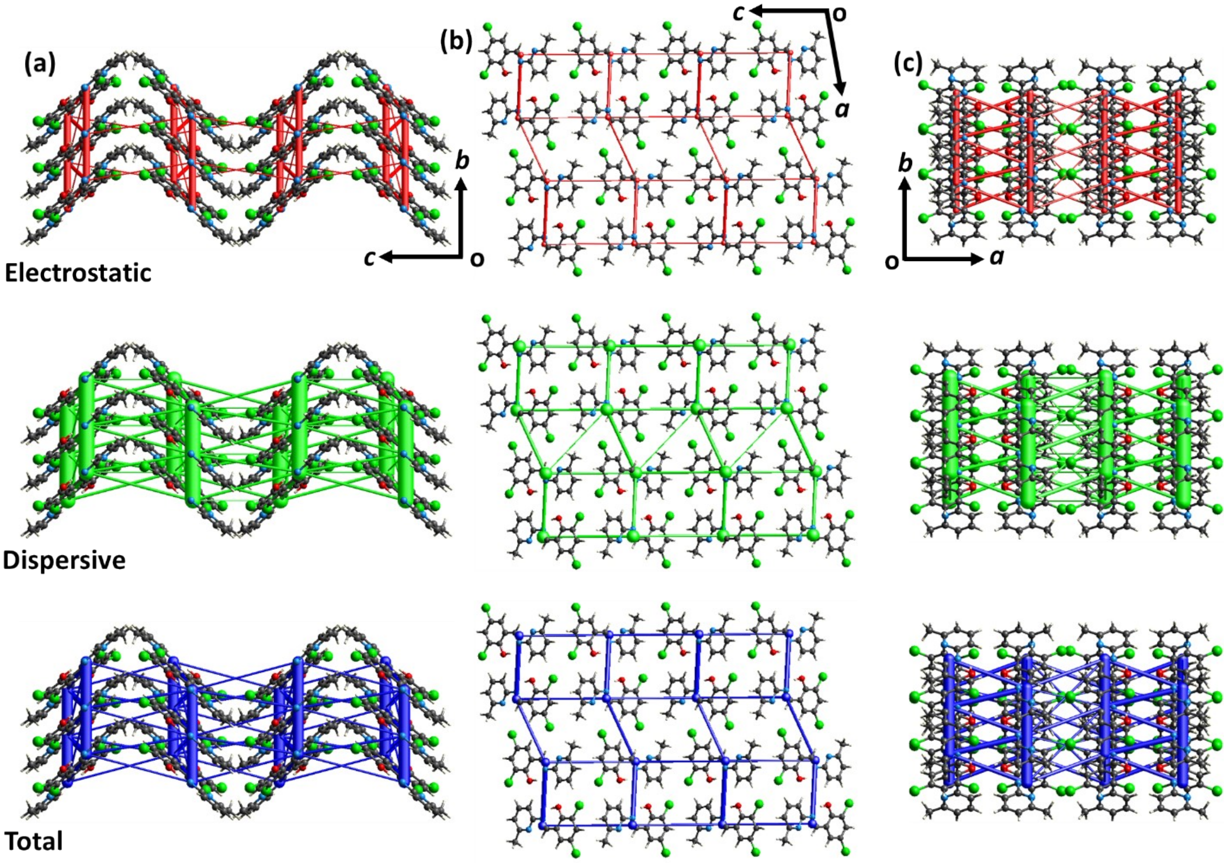

Fig. S3.5| Visualization of energy frameworks showing electrostatic (top, red), dispersion (middle, green) components and total interaction energy (below, blue) for DMP, in the (a) (100), (b) (001) and (c) (010) faces, respectively. The energy scale factor is 25 and the energy threshold is 5 kJ.mol^{-1}.

Table S3.3| Molecular structure pairs and the interaction energies (kJ.mol^{-1}) obtained from energy frameworks calculation for DMP. Scale factors are in the lower table.

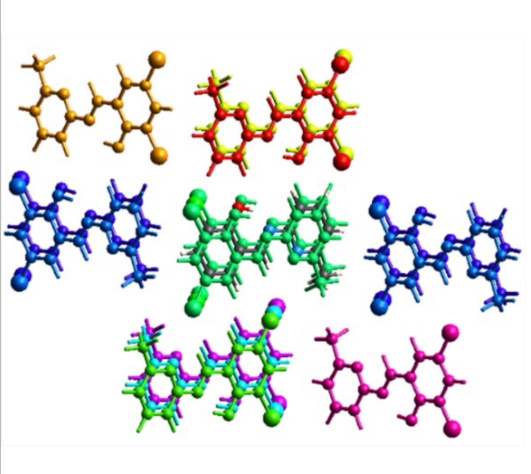

Interaction Energies (kJ/mol)
R is the distance between molecular centroids (mean atomic position) in Å.

Total energies, only reported for two benchmarked energy models, are the sum of the four energy components, scaled appropriately (see the scale factor table below)

	N	Symop	R	Electron Density	E_ele	E_pol	E_dis	E_rep	E_tot
	1	-x, -y, -z	7.24	B3LYP/DGDZVP	-14.1	-2.4	-18.4	20.4	-20.1
	1	-x, y, -z+1/2	10.79	B3LYP/DGDZVP	0.2	-0.1	-3.7	7.7	1.6
	1	-x, -y, -z	7.97	B3LYP/DGDZVP	-17.0	-4.5	-21.7	20.7	-27.5
	1	-x, -y, -z	8.84	B3LYP/DGDZVP	-5.6	-0.5	-10.3	8.8	-9.9
	2	x, y, z	4.28	B3LYP/DGDZVP	-31.1	-1.3	-84.7	89.1	-52.5
	1	-x, -y, -z	7.26	B3LYP/DGDZVP	-9.3	-1.2	-23.0	21.9	-17.2
	2	x, -y, z+1/2	10.04	B3LYP/DGDZVP	-4.1	-0.5	-14.9	12.7	-9.9
	2	x, -y, z+1/2	10.59	B3LYP/DGDZVP	-6.2	-0.9	-15.0	13.3	-12.0
	1	-x, -y, -z	7.99	B3LYP/DGDZVP	-4.1	-0.4	-7.8	6.1	-7.6
	1	-x, y, -z+1/2	11.45	B3LYP/DGDZVP	-1.0	-0.1	-6.8	6.9	-2.8

Scale factors for benchmarked energy models
See Mackenzie et al. IUCrJ (2017)

Energy Model	k_ele	k_pol	k_disp	k_rep
CE-HF ... HF/3-21G electron densities	1.019	0.651	0.901	0.811
CE-B3LYP ... B3LYP/6-31G(d,p) electron densities	1.057	0.740	0.871	0.618

S4 Mechanical Flexibility of DMP

S4.1 Three Point Bending| Three-point bending experiments were conducted using a pair of forceps and a needle. The crystal was stabilized on the glass slide (i.e. to avoid jumping) by placing it in a small drop of Fomblin oil. Bending over the (00$\bar{1}$) face, **Fig. S4.1**, revealed elastoplastic bending. At low loadings, the deformation was fully reversible, but became irreversible at higher loadings (e.g. **Fig. S4.1o**).

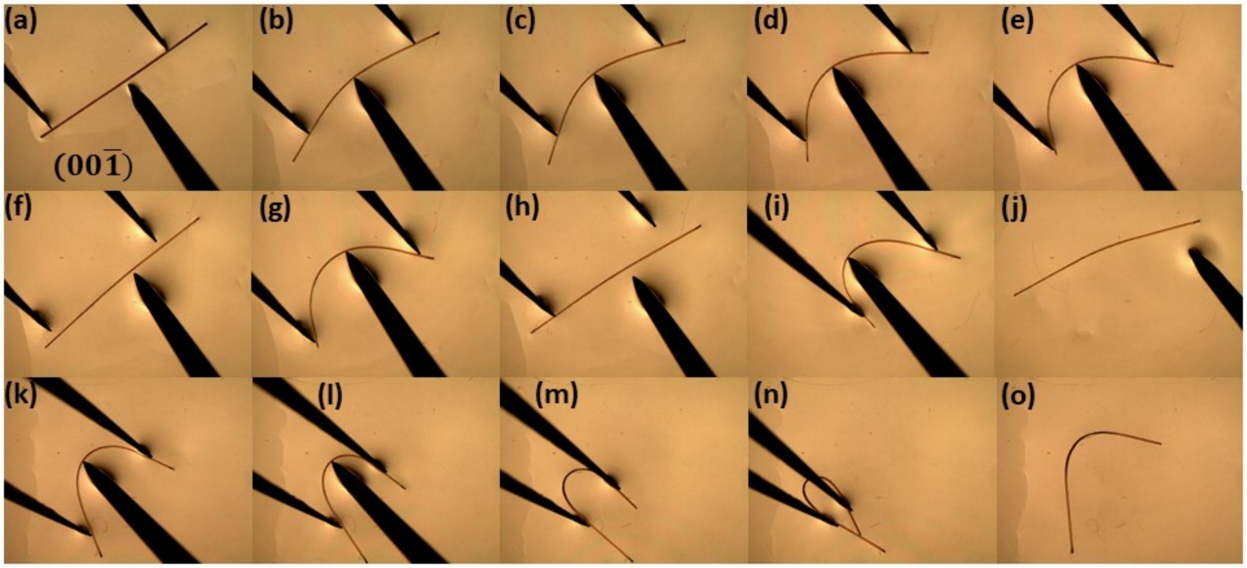

Fig. S4.1| a-o) Optical microscopic photographs of single crystals of DMP undergoing three-point bending along the crystallographic (100)/($\bar{1}$00) face: a-j) Elastic bending: the crystal regains its pristine shape after withdrawal the stress. k-o) Plastic deformation: irreversible mechanical deformation on the same crystal by increasing stress.

In contrast, three-point bending over the (100) face, **Fig. S4.2**, shows a small degree of macroscopic elasticity at low deforming loads, followed by partial fracture of the convex surface at higher loadings. Interestingly, the unfractured crystal remains flexible (see **Fig. S4.2 e-f**). Following from the apparent self-healing of DMP crystals during AFM deformation over this face, we suspect the apparent elasticity stems from self-healing of microfracture on the (100) face (see discussion of the atomic force microscopy results in the main manuscript)

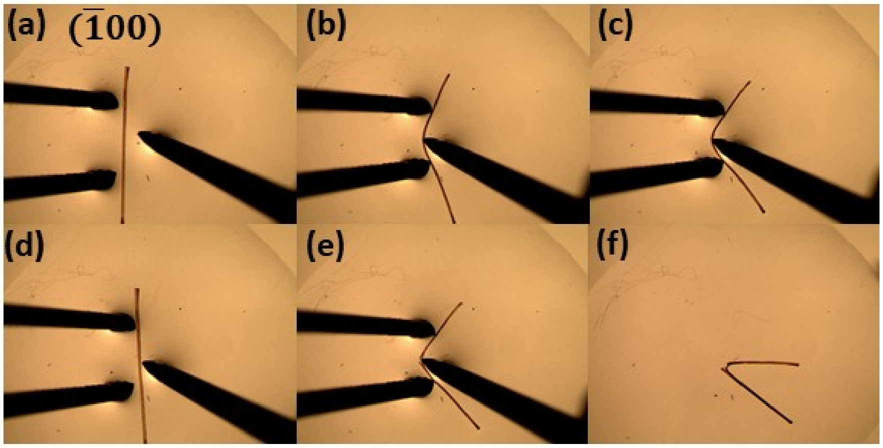

Fig. S4.2| a-f) Optical microscopic photographs of single crystals of DMP (($\bar{1}$00) face) undergoing three-point bending along the crystallographic (001)/(001−) face: a-c) Elastic bending: the crystal regains its pristine shape after withdrawal the stress. i-k) Brittle fracture: crystal broke into pieces by increasing stress.

S4.2 Calculation of Elastic Strain| Using microscopic images of bending, **Fig. 4.3**, the elastic strain of elastically bent single crystals of DMP were calculated according to the Euler-Bernoulli equation, which relates the beam thickness, *t* and radius of curvature, *r=d/2*, to elastic strain,

$$\epsilon = \frac{t}{d} = \frac{0.07\ mm}{2.86\ mm} = 0.0245$$

Hence, the elastic strain observed for DMP is 2.45 %

(a)

(b)

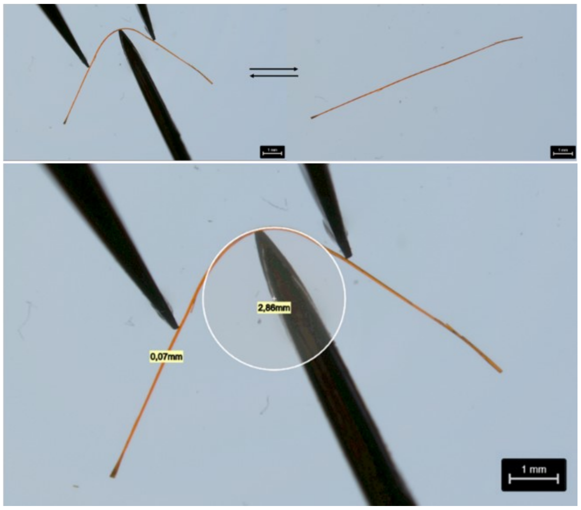

Fig. 4.3| Measurement of the elastic strain in a single crystal of DMP. (a) Demonstration of the reversibility of deformation in the single crystal, confirming measurements were made in the elastic regime of bending. (b) Measurement of the crystal thickness and diameter of bending.

S5 Thermochromic Behaviour of DMP

o-Hydroxy Schiff base molecules are well-known to exhibit thermochromism, resulting from intramolecular proton transfer. Small amounts of DMP crystals were put into glass test tubes which were then submerged into liquid nitrogen. DMP crystals changed colour from orange to yellow, and upon removal from liquid nitrogen the crystal return to their original orange colour, **Fig. S5.1.** To further study the reversible thermochromism of DMP crystal, we controlled the temperature of the crystal by using a Linkam LST 420 hot-stage. The videos were collected between 20 °C and -170 °C under a NikonAZ100 MULTIZOOM microscope with a DS-Ri1 12.7 megapixel camera. The crystal started to change colour from orange to light orange to yellow (**Fig. S5.2**; **Video S3**). Upon re-heating the crystal to 20 °C, the crystal returned to its original orange colour (**Fig. S5.2**; **Video S3**). A plastically bent crystal also showed the same reversible thermochromism over similar controlled temperature conditions (**Fig. S5.3**; **Video S4**). To verify if the temperature affects the elasticity, we first cooled a metal plate by adding excess liquid nitrogen. The crystal was placed on the cooled plate (became visibly yellow) and bent around a metal rod using wooden sticks. The elasticity of DMP crystals was also observed at low temperature with distinct colour change from orange to yellow. The crystal could be bent repeatedly under the low temperature condition (**Video S3**).

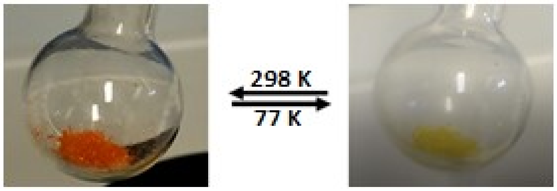

Fig. S5.1| Thermochromism exhibited by single crystals of DMP crystals at 77 K and 298 K, respectively.

[illegible] microscope

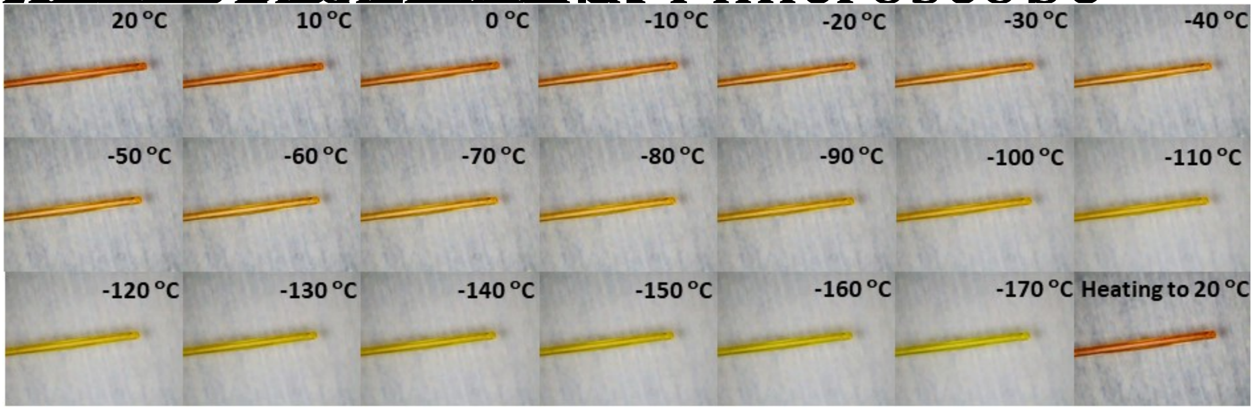

Fig. S5.2| Optical microscope images of straight single crystal of DMP at different temperatures during colling from 20 °C to -170 °C (interval 10 °C), and after reheating again to 20 °C showing reversible thermochromism.

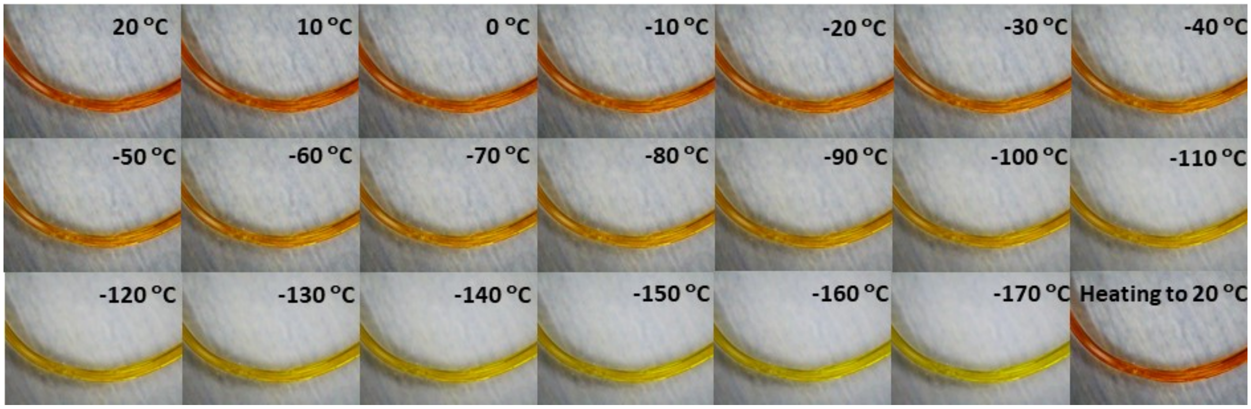

Fig. S5.3| Optical microscope images of plastically bent crystal of DMP at different temperatures during colling from 20 °C to -170 °C (interval 10 °C), and after reheating again to 20 °C showing reversible thermochromism.

Importantly, crystal structure analysis revealed that no structural transition occurred in DMP across the thermochromic transition (see **Section S3**), suggesting the mechanical properties could be largely preserved at low temperature. Moreover, the thermochromic behaviour of these crystals appeared to be unaffected by bending, with plastically bent crystals transitioning continuously and reversibly from orange to yellow across the same temperature range as the straight crystals, **Fig. S5.3**. Our results therefore suggest that flexible o-hydroxy Schiff base crystals may be suitable for applications as flexible thermal-sensors or thermo-optical devices and will be explored in a detailed follow up investigation.

S6 Force-Displacement Curves – Atomic Force Microscopy

Force-displacement curves (FDCs) for a crystal of DMP were measured by AFM with different maximum loads as shown in **Fig. S6.1** (red markers F_{max} = 1.5 μN and brown markers F_{max}= 3.5 μN). Up to an applied load of 1.5μN both experimental curves are in very good agreement with a fit by the Hertz theory (black dashed line) which describes the elastic deformation of a homogeneous material. From this fit the Young's modulus of the sample can be estimated as E =5.7 GPa (with Poisson ratio ν = 0.3). However, for higher applied forces > 1.5μN (brown markers) a characteristic deviation from the fit is observed, which is due to yield, *i.e.* plastic deformations. This can also be seen in the hysteresis of the approach (full markers) and retraction (empty markers) part of the FDC, as well as in the offset of the displacement D at F = 0. For applied forces < 1.5 μN the hysteresis is small ($\alpha_{hyst} = 7.2 \times 10^{-16}$ Nm) and the deformation recovers nearly completely (dD < 1 nm), which is consistent with elastic deformations. However, for applied forces > 1.5 μN the hysteresis is significantly higher (($\alpha_{hyst} = 2.5 \times 10^{-14}$ Nm) and deformations do not recover (dD = 9nm).

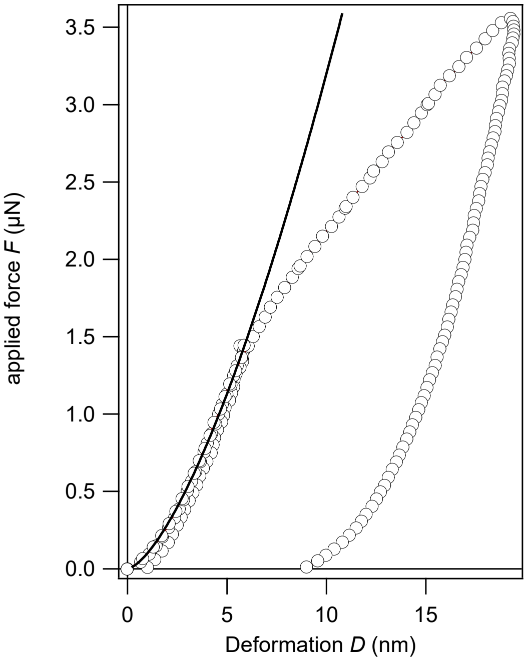

Fig. S6.1| Comparison of the force displacement curves measured by atomic force microscopy on the (A) (001) crystallographic face, and (B) (100) crystallographic face.

S7 Synchrotron Based Microfocus X-Ray Diffraction

Typical laboratory X-ray diffraction uses a beam size on the order of 100-1000 μm in diameter. Correspondingly, resolving unit cell geometry across the bent positions of a single crystal is impeded by averaging over a large spatial distribution. To overcome this challenge we have used a micro-focus beam (0.922 × 3.67 μm beam size) available at the SPring-8 synchrotron facility (BL40XU beamline) to capture the spatially resolved evolution of unit cell geometry across each of the elastically and plastically flexible crystals, **Fig.s 7.1** and **7.2**, respectively.

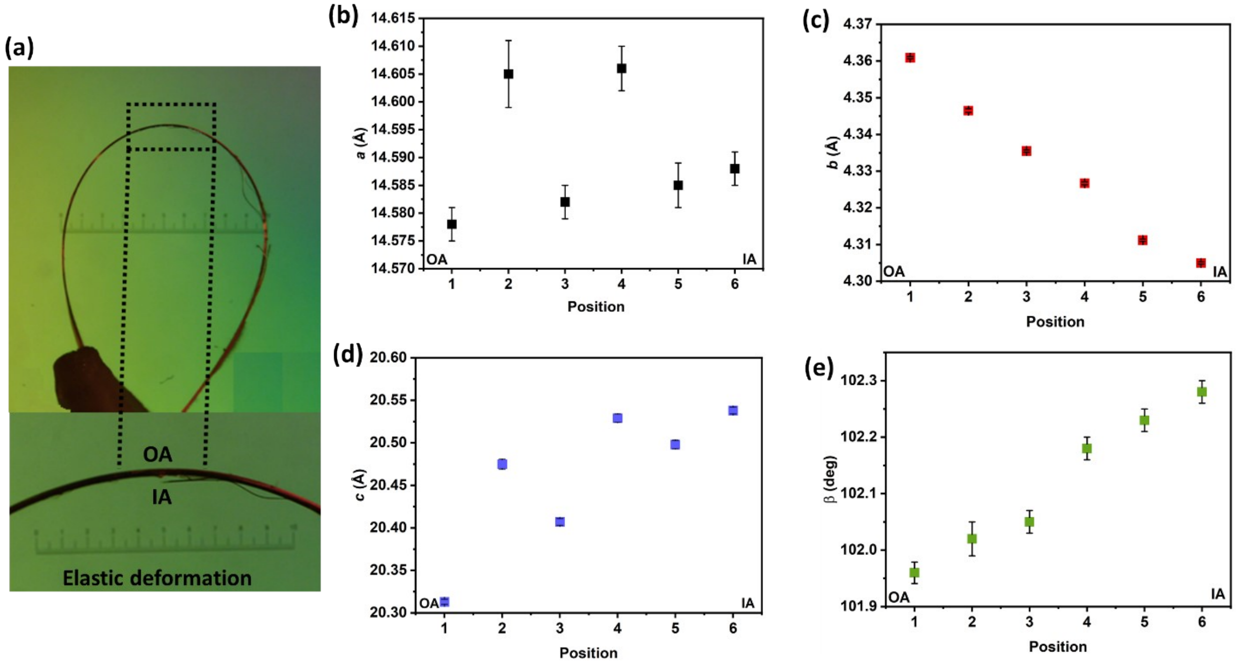

Fig. S7.1| Microfocus synchrotron X-ray single crystal diffraction of an elastically bent crystal of DMP. (a) Microphotograph of the elastically bent single crystal, with indications of the outer arc (OA) and inner arc (IA) Note the major ruler ticks indicate 1.33 mm. Diffraction data were collected at equidistant steps (50 μm) across the crystal from OA to IA, with the corresponding unit cell parameters shown for each position in (b) a-axis; (c) b-axis; (d) c-axis, and (e) monoclinic β angle. Estimated standard deviations are shown for each data point.

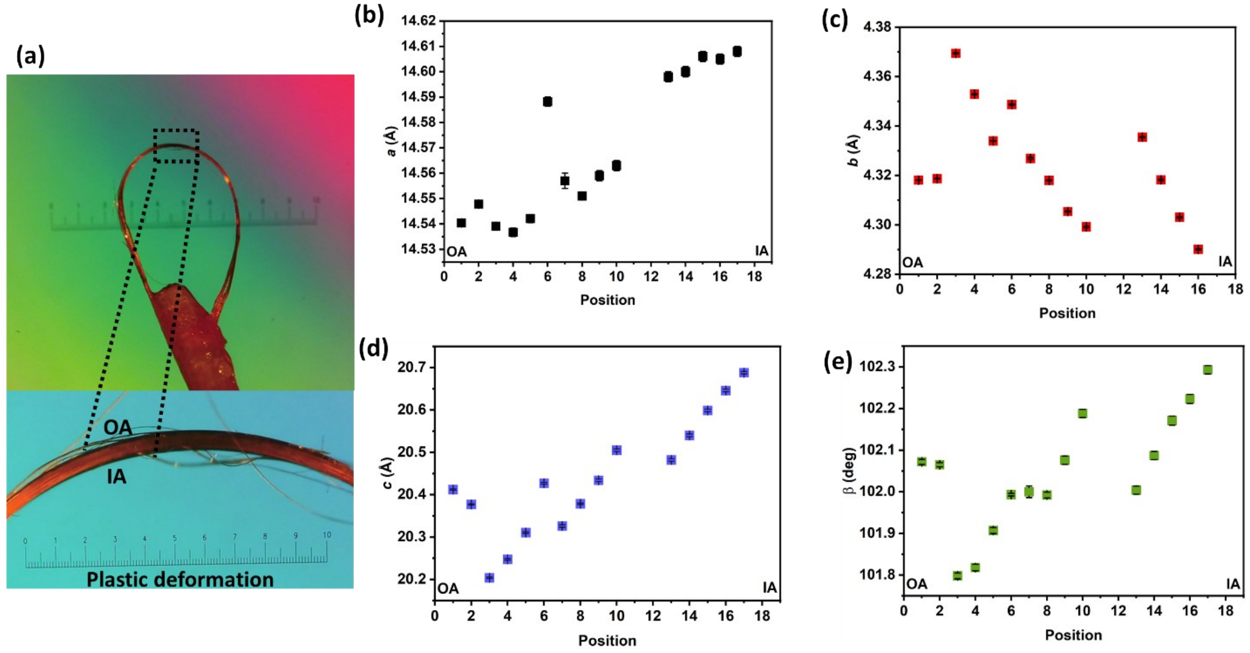

Fig. S7.2| Microfocus synchrotron X-ray single crystal diffraction of a plastically bent crystal of DMP. (a) Microphotograph of the elastically bent single crystal, with indications of the outer arc (OA) and inner arc (IA) Note the major ruler ticks indicate 1.33 mm. Diffraction data were collected at equidistant steps (50 μm) across the crystal from OA to IA, with the corresponding unit cell parameters shown for each position in (b) a-axis; (c) b-axis; (d) c-axis, and (e) monoclinic β angle. Estimated standard deviations are shown for each data point.

S8 Vibrational Spectroscopy of DMP

S8.1 Simulated Vibrational Frequencies| DMP crystallises in monoclinic space group $P2/c$ (point group C_{2h}). With 112 atoms in the primitive cell, there are 336 normal modes of vibration $\Gamma_{acoustic} = 2B_u + A_u$ and $\Gamma_{optical} = 84\ A_g + 83\ A_u + 84\ B_g + 82\ B_u$. Both the A_g and B_g modes are Raman active. The absence of imaginary (negative) frequencies confirms that our simulated structure corresponds to an energetic minimum on the potential energy surface.

Table S8.1 | Simulated Γ point vibrational frequencies for DMP alongside irreducible representation (I.R.), calculated at PBE-D2 level of theory.

M	I.R.	ν /cm^{-1}	M	I.R.	ν /cm^{-1}	M	I.R.	ν /cm^{-1}	M	I.R.	ν /cm^{-1}	M	I.R.	ν /cm^{-1}
**1**	Bu	0	**51**	Bu	162.549	**101**	Bu	509.880	**151**	Ag	793.992	**201**	Bu	1075.377
**2**	Bu	0	**52**	Ag	163.301	**102**	Bg	510.182	**152**	Au	794.643	**202**	Bg	1075.506
**3**	Au	0	**53**	Ag	186.140	**103**	Au	510.310	**153**	Bg	836.661	**203**	Ag	1075.905
**4**	Bu	25.143	**54**	Bg	187.623	**104**	Bu	510.316	**154**	Ag	837.456	**204**	Au	1076.036
**5**	Bg	27.837	**55**	Bu	190.301	**105**	Ag	510.600	**155**	Bu	839.149	**205**	Ag	1088.497
**6**	Bu	31.673	**56**	Au	190.768	**106**	Au	511.362	**156**	Au	840.137	**206**	Bg	1088.779
**7**	Bg	32.372	**57**	Bu	195.588	**107**	Ag	511.621	**157**	Bu	848.368	**207**	Bu	1091.066
**8**	Au	33.081	**58**	Bg	197.760	**108**	Bg	512.119	**158**	Au	848.657	**208**	Au	1091.125
**9**	Ag	35.096	**59**	Au	197.784	**109**	Bu	525.626	**159**	Ag	858.261	**209**	Ag	1137.094
**10**	Au	40.394	**60**	Ag	199.510	**110**	Bg	526.910	**160**	Bg	858.672	**210**	Bg	1137.286
**11**	Ag	40.396	**61**	Bu	212.297	**111**	Ag	529.342	**161**	Bu	864.035	**211**	Au	1137.958
**12**	Bg	46.340	**62**	Bg	213.575	**112**	Au	529.376	**162**	Au	864.739	**212**	Bu	1138.167
**13**	Bg	47.582	**63**	Au	214.386	**113**	Bg	545.382	**163**	Ag	865.406	**213**	Ag	1162.200
**14**	Au	49.608	**64**	Ag	214.624	**114**	Bu	545.539	**164**	Bg	866.129	**214**	Bg	1162.438
**15**	Bu	50.112	**65**	Ag	238.949	**115**	Au	547.159	**165**	Bg	886.670	**215**	Bu	1163.000
**16**	Au	52.745	**66**	Bu	239.396	**116**	Ag	547.760	**166**	Ag	886.838	**216**	Au	1167.102
**17**	Ag	52.797	**67**	Bg	239.583	**117**	Bu	556.295	**167**	Au	886.925	**217**	Bg	1189.174
**18**	Bg	55.750	**68**	Au	239.612	**118**	Au	556.776	**168**	Bu	886.936	**218**	Ag	1189.848
**19**	Ag	57.408	**69**	Au	296.510	**119**	Bg	560.030	**169**	Bg	931.018	**219**	Bu	1191.792
**20**	Bg	58.793	**70**	Bg	296.563	**120**	Ag	560.035	**170**	Bu	931.348	**220**	Au	1192.783
**21**	Ag	64.870	**71**	Bu	297.031	**121**	Bu	593.902	**171**	Ag	935.335	**221**	Ag	1217.502
**22**	Ag	66.092	**72**	Ag	297.159	**122**	Au	594.264	**172**	Au	936.301	**222**	Bg	1217.580
**23**	Ag	67.724	**73**	Bu	304.920	**123**	Bg	594.533	**173**	Bu	951.865	**223**	Bu	1217.978
**24**	Au	69.436	**74**	Au	306.345	**124**	Ag	594.908	**174**	Bg	952.894	**224**	Au	1219.701
**25**	Bu	70.492	**75**	Bg	307.143	**125**	Bg	602.126	**175**	Au	953.222	**225**	Bu	1252.342
**26**	Bg	72.421	**76**	Ag	308.771	**126**	Bu	603.059	**176**	Ag	955.290	**226**	Bg	1252.639
**27**	Bu	76.955	**77**	Bg	313.874	**127**	Au	604.769	**177**	Au	969.293	**227**	Ag	1252.864
**28**	Au	80.938	**78**	Bu	314.219	**128**	Ag	605.575	**178**	Bu	969.300	**228**	Au	1253.463
**29**	Ag	81.255	**79**	Au	314.320	**129**	Bg	676.800	**179**	Bg	969.488	**229**	Au	1275.356
**30**	Bg	82.977	**80**	Ag	314.447	**130**	Au	677.106	**180**	Ag	969.527	**230**	Bu	1275.898
**31**	Au	86.445	**81**	Bg	360.996	**131**	Bu	677.404	**181**	Bu	977.980	**231**	Bg	1276.524
**32**	Bu	87.490	**82**	Ag	361.674	**132**	Ag	677.643	**182**	Bg	978.047	**232**	Ag	1277.267
**33**	Bu	98.077	**83**	Bu	363.830	**133**	Bu	721.080	**183**	Au	978.600	**233**	Bg	1294.726
**34**	Bg	100.309	**84**	Au	364.084	**134**	Ag	721.530	**184**	Ag	978.636	**234**	Ag	1295.441
**35**	Ag	102.773	**85**	Bu	371.679	**135**	Au	721.812	**185**	Bg	995.951	**235**	Au	1296.198
**36**	Au	103.114	**86**	Bg	371.930	**136**	Bg	722.201	**186**	Bu	996.191	**236**	Bu	1296.727
**37**	Au	104.849	**87**	Ag	371.945	**137**	Bu	725.924	**187**	Ag	996.663	**237**	Au	1318.928
**38**	Ag	105.054	**88**	Au	372.343	**138**	Bg	726.190	**188**	Au	997.002	**238**	Bg	1318.961
**39**	Bu	108.268	**89**	Bu	380.733	**139**	Ag	727.884	**189**	Bu	1007.926	**239**	Bu	1319.146
**40**	Bg	111.068	**90**	Au	381.301	**140**	Au	728.447	**190**	Bg	1007.942	**240**	Ag	1319.289
**41**	Bg	143.717	**91**	Bg	382.492	**141**	Au	739.192	**191**	Au	1016.881	**241**	Bg	1335.879
**42**	Ag	144.287	**92**	Ag	383.370	**142**	Bu	739.455	**192**	Bu	1017.183	**242**	Ag	1336.173
**43**	Bu	145.581	**93**	Bu	404.990	**143**	Bg	739.506	**193**	Ag	1018.099	**243**	Bu	1337.268
**44**	Au	145.590	**94**	Bg	405.945	**144**	Ag	740.432	**194**	Bg	1018.331	**244**	Au	1338.141
**45**	Bg	155.038	**95**	Au	406.427	**145**	Bg	776.452	**195**	Ag	1020.431	**245**	Bu	1344.593
**46**	Ag	156.471	**96**	Ag	407.510	**146**	Ag	776.932	**196**	Au	1020.897	**246**	Bg	1344.988
**47**	Bu	156.886	**97**	Bu	432.306	**147**	Bu	777.330	**197**	Bu	1021.203	**247**	Ag	1355.398
**48**	Au	157.370	**98**	Bg	432.430	**148**	Au	778.436	**198**	Bg	1022.270	**248**	Au	1356.376
**49**	Bg	158.775	**99**	Au	433.152	**149**	Bu	791.808	**199**	Au	1022.681	**249**	Bg	1384.963
**50**	Au	159.280	**100**	Ag	433.451	**150**	Bg	793.390	**200**	Ag	1023.511	**250**	Ag	1385.041

M	I.R.	ν /cm^{-1}	M	I.R.	ν /cm^{-1}	M	I.R.	ν /cm^{-1}	M	I.R.	ν /cm^{-1}	M	I.R.	ν /cm^{-1}
**251**	Au	1386.369	**301**	Bu	2939.766									
**252**	Ag	1386.624	**302**	Bg	2939.806									
**253**	Bu	1386.993	**303**	Ag	2942.093									
**254**	Au	1387.958	**304**	Au	2942.096									
**255**	Bu	1389.016	**305**	Bu	3018.521									
**256**	Bg	1389.063	**306**	Bg	3018.583									
**257**	Au	1412.081	**307**	Ag	3018.955									
**258**	Ag	1412.475	**308**	Au	3018.974									
**259**	Bg	1413.238	**309**	Ag	3036.467									
**260**	Ag	1414.234	**310**	Bg	3036.489									
**261**	Bu	1414.289	**311**	Au	3036.510									
**262**	Au	1416.398	**312**	Bu	3036.536									
**263**	Bu	1417.929	**313**	Bu	3049.287									
**264**	Bg	1419.768	**314**	Bg	3049.288									
**265**	Bu	1431.446	**315**	Ag	3052.751									
**266**	Bg	1432.568	**316**	Au	3052.757									
**267**	Au	1433.286	**317**	Ag	3117.021									
**268**	Ag	1433.917	**318**	Bg	3117.193									
**269**	Bg	1436.853	**319**	Au	3117.412									
**270**	Bu	1437.539	**320**	Bu	3117.551									
**271**	Ag	1438.678	**321**	Ag	3122.666									
**272**	Au	1439.452	**322**	Bu	3122.692									
**273**	Bu	1472.291	**323**	Au	3122.833									
**274**	Au	1473.635	**324**	Bg	3122.936									
**275**	Ag	1474.946	**325**	Bg	3124.637									
**276**	Bg	1475.630	**326**	Ag	3124.667									
**277**	Bg	1528.008	**327**	Bu	3125.445									
**278**	Ag	1528.716	**328**	Au	3125.509									
**279**	Bu	1529.495	**329**	Ag	3130.499									
**280**	Au	1531.865	**330**	Bg	3130.601									
**281**	Au	1559.125	**331**	Au	3130.875									
**282**	Bu	1559.971	**332**	Bu	3130.992									
**283**	Bg	1560.655	**333**	Bu	3132.534									
**284**	Ag	1561.221	**334**	Au	3132.542									
**285**	Bg	1568.145	**335**	Ag	3141.481									
**286**	Bu	1568.873	**336**	Bg	3141.484									
**287**	Ag	1569.581												
**288**	Au	1570.137												
**289**	Bu	1584.270												
**290**	Ag	1584.800												
**291**	Au	1584.954												
**292**	Bg	1585.306												
**293**	Bu	1605.148												
**294**	Au	1606.490												
**295**	Ag	1608.705												
**296**	Bg	1609.141												
**297**	Ag	2339.256												
**298**	Bg	2340.123												
**299**	Bu	2345.269												
**300**	Au	2351.840												

S8.2 Experimental Raman Spectra| The effects of mechanical bending on DMP single crystals were analysed by μ-focus Raman spectroscopy to explore the potential influence of bending on molecular deformation, **Fig. 8.1**. For the same elastically bent single crystal, **Fig. 8.1b** and **8.1d**, the Raman spectra at the inner (IA), middle (MA), and outer arc (OA) remain effectively identical, with no detectable shift in the positions of the vibrational bands, **Table S8.2**. This strongly suggests that no significant perturbations on the molecular structure occur during elastic bending. We do note minor changes in the relative intensities of the Raman bands as compared with the straight portion (SP) of the elastically bend crystal and a separate single crystal (S), which presumably result from a change in the orientation of the crystal in the polarization direction of the laser, or due to formation of defects during bending. The same effect is observed in plastically bent single crystals of DMP, **Fig. 8.1c, Fig. 8.1e**, and **Table S8.3,** again highlighting that no major molecular level deformations occur during bending. We note that comparison of intensities between different crystals is precluded by different concentration of defects and microstructure, leading to difficulties in extracting meaning when comparing the intensities of data collected from straight crystals, elastically bent crystals, and plastically bent crystals. The only notable difference in the Raman spectra of the plastically bent crystals is the apparent splitting of the Raman band at *ca.* 190 cm^{-1} (M4 in Table S8.2). This effect is presumably due to the anisotropic strain causing resolution of closely spaced vibrational bands (see Table S8.1) in DMP.

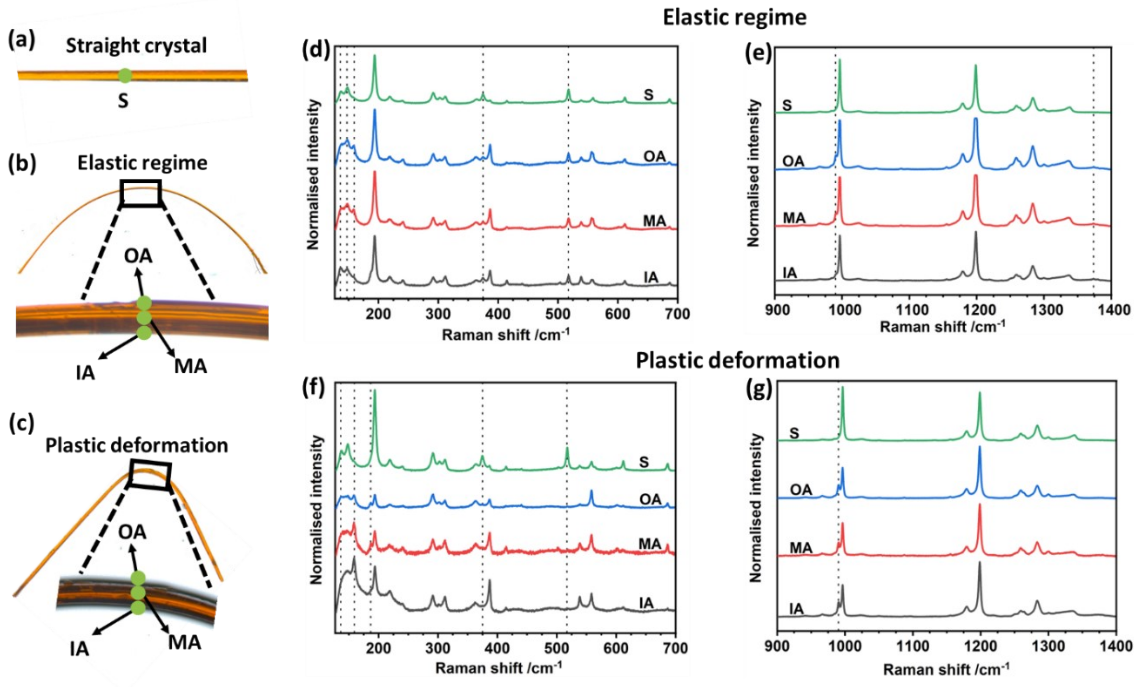

Fig. S8.1| μ-Focus Raman spectra for the straight, elastically and plastically bent crystals of DMP over the range 150-1650 cm^{-1}. (a) Photograph of straight crystal. (b) Photograph of an elastically bent crystal with positions of data collection at outer arc (OA), middle arc (MA), inner arc (IA) and straight part (SP). (c) Photograph of a plastically bent crystal with positions of data collection at outer arc (OA), middle arc (MA), inner arc (IA) and straight part (SP). (d, e) Comparison of normalized Raman spectra collected at OA, MA and IA of an elastically bent crystal, as compared with the spectrum collected from the straight crystal (S in (a)). (f, g) Comparison of normalized Raman spectra collected at OA, MA, IA, and SP of the plastically bent crystal, as compared with the spectrum collected from the straight crystal and straight crystal (S in (a)).

Table S8.1| Peaks in the Raman spectra of elastically bent DMP for frequencies < 500 cm^{-1} measured for the inner arc (IA), middle arc (MA) and outer arc (OA), as denoted in **Figure S8.1**.

	Raman Frequencies /cm^{-1}			
	IA	MA	OA	Note
**M1**	137.065	137.736	137.736	
**M2**	148.147	148.147	148.147	
**M3**	158.199	158.533	158.533	Large increase in intensity as IA<MA<OA
**M4**	193.546	193.750	193.713	
**M5**	218.729	219.059	219.72	
**M6**	240.487	240.815	240.158	
**M7**	291.836	291.510	291.510	
**M8**	302.529	302.555	3011.907	
**M9**	311.959	311.632	311.309	
**M10**	363.468	363.468	362.828	
**M11**	374.02	374.658	374.658	
**M12**	386.457	386.457	386.457	
**M13**	414.630	414.629	414.308	
**M14**	439.932	439.932	440.57	

Table S8.2| Peaks in the Raman spectra of plastically bent DMP for frequencies < 500 cm^{-1} measured for the inner arc (IA), middle arc (MA) and outer arc (OA), as denoted in **Figure S8.1**. Note the Raman spectra from the plastically bent crystal have significantly higher background than from the elastically bent crystal, making the identification of small peaks (e.g. M1, M11 and M14) difficult to identify in some cases.

	**Raman Frequencies /cm^{-1}**			
	IA	MA	OA	Note
**M1**	--	--		Note band at *ca.* 137 cm^{-1} is not visible above background.
**M2**	146.805	147.810	148.147	
**M3**	158.868	158.533	159.202	
**M4**	188.560<br>193.546	186.565<br>192.883	186.898<br>193.546	
**M5**	218.398	217.737	219.059	
**M6**	240.158	240.487	239.829	
**M7**	291.186	291.186	291.186	
**M8**	301.258	301.258	301.583	
**M9**	311.956	311.632	311.632	
**M10**	363.148	363.788	362.508	
**M11**	--	374.978	374.028	

**M12**	386.776	386.457	386.457	
**M13**	414.630	414.630	413.346	
**M14**	--	--	439.932	Note the band at ca. 440 cm^{-1} is generally not visible above background.

S9 Anisotropic Compression of DMP

To assess the effects of anisotropic mechanical deformation on the structure of DMP we imposed anisotropic compression/tension over the unit cell. To mimic the direction of compression / tension experienced during elastic bending, expansion and contraction of the unit cell was conducted along the b-axis (*i.e.* the long axis of the crystal). The b-axis was held constant at the given strain value ($\xi = 100 \times (b - b_0)/b_0$) and the rest of the unit cell was allowed to relax in response to the perturbation, **Table S9.1**. We note that the expansion / contraction of the b-axis was exaggerated beyond the stresses typically observable in molecular crystals. This was done to emphasis the structural response to bending. It is worth highlighting that despite applied strains ranging nearly 10%, the crystal volume varies by only *ca.* 3.5%. This demonstrates the relaxation of the crystal unit cell in directions orthogonal to the applied strain as being critical to the material response. This is consistent with experimental observation from μ-focus XRD (e.g. Fig. 3 in the main manuscript).

Table S9.1| Effect of anisotropic distortion along the b-axis on the unit cell of DMP, determined from PBE-D2 DFT simulations. Note that the value of strain is defined as $\xi = 100 \times (b - b_0)/b_0$.

$\Delta\beta$ /Å	ξ/%	**a /Å**	**b /Å**	**c /Å**	β	**V**	Δ**Energy /eV**
+0.2	4.670	14.207	4.384	19.105	99.236	1174.454	13.584
+0.1	2.335	14.207	4.384	19.107	99.244	1174.532	3.176
+0.09	2.125	14.211	4.374	19.154	99.308	1174.819	2.854
+0.08	1.891	14.214	4.364	19.183	99.341	1174.037	2.133
+0.07	1.658	14.215	4.354	19.213	99.383	1173.146	1.778
+0.06	1.424	14.217	4.344	19.245	99.427	1172.444	1.449
+0.05	1.191	14.220	4.334	19.275	99.462	1171.670	0.966
+0.04	0.957	14.223	4.324	19.302	99.493	1170.693	0.595
+0.03	0.724	14.225	4.314	19.328	99.525	1169.680	0.321
+0.02	0.467	14.227	4.304	19.363	99.574	1169.049	0.220
+0.01	0.233	14.230	4.294	19.391	99.607	1168.140	0.067
0	0	14.232	4.283	19.421	99.656	1167.016	0
-0.01	-0.210	14.234	4.274	19.447	99.676	1166.186	0.046
-0.02	-0.444	14.237	4.264	19.476	99.714	1165.266	0.135
-0.03	-0.677	14.240	4.254	19.514	99.753	1164.879	0.435
-0.04	-0.911	14.242	4.244	19.545	99.791	1164.075	0.722
-0.05	-1.144	14.244	4.234	19.570	99.831	1162.831	0.772
-0.06	-1.378	14.246	4.224	19.614	99.886	1162.658	1.145
-0.07	-1.611	14.248	4.214	19.640	99.916	1161.502	1.614
-0.08	-1.845	14.251	4.204	19.665	99.946	1160.368	2.189
-0.09	-2.078	14.254	4.194	19.693	99.976	1159.371	2.989
-0.1	-2.311	14.255	4.184	19.734	100.041	1158.865	3.060
-0.2	-4.646	14.280	4.084	20.056	100.455	1150.155	9.176
-0.3	-6.981	14.303	3.984	20.359	100.843	1139.317	20.560
-0.4	-9.316	14.326	3.884	20.659	101.212	1127.502	40.544

Accompanying the adjustment of unit cell dimensions, anisotropic compression leads to an increase on the internal energy of the unit cell, **Fig. S9.1.** This strain energy represents the excess energy held by the stressed crystal, i.e. the energy the crystal will aim to alleviate through deformation such as delamination (see **Section S**). From μ-focus XRD, a strain of *ca.* 2% is expected in elastically bent crystals of DMP (**Fig.**

3 in main text), which is consistent with the calculated elastic strain calculation suggestion of $\epsilon \approx 2.5\%$ (**ESI S4**). According to our DFT models (PBE-D2), this degree of strain is accompanied by a *ca.* 3- kJ.mol^{-1} increase in the internal energy of system. These energies are well within reach of standard experimental procedures and thermal energy at room temperature (*ca.* 2.5 kJ.mol^{-1}), and are on par with the energies that separate normal solid-state processes and weak intermolecular interactions. It is therefore very reasonable to assume that these energies are achieved upon bending without causing significant damage to the system.

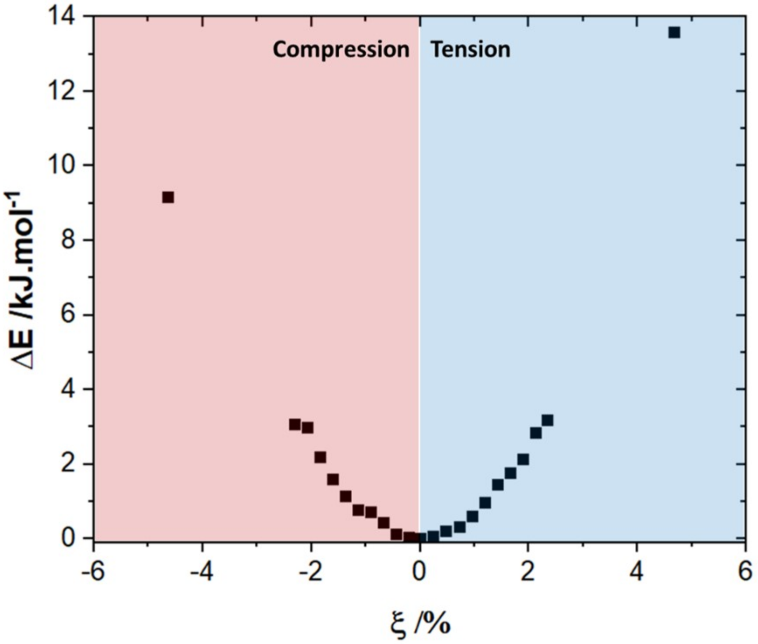

Fig. S9.1| Increase in unit cell internal energy as a function of anisotropic compression for DMP, obtained at PBE-D2 level of theory.

S10 Crystallographic Structure of 4-bromo-6-[(6-chloropyridin-2-ylimino)methyl]phenol (CMP)

S10.1 Crystal packing of CMP| As a second example of a mechanically flexible herringboned molecular crystal, we compare DMP to our previously reported 4-bromo-6-[(6-chloropyridin-2-ylimino)methyl]phenol (CMP), **Fig. S10.1**.[12] CMP crystalizes in the monoclinic space group $P2_1/c$ with one CMP molecule in the asymmetric unit. Crystal face indexing indicated that the (010)/(0$\bar{1}$0) and (001)/(00$\bar{1}$) faces are the major faces, with the minor face being the (100)/($\bar{1}$00). In the crystal packing CMP molecules close pack in orthogonal directions through their weak hydrophobic –Cl groups to form zig-zag chains along the *b*-axis. The chains are connected *via* C–H···O, C–H···Cl and C–H···Br interactions along the crystallographic *c*-axis. These chains are further slip-stacked *via* π···π interactions along the growth axis of the crystal (*a*-axis) to construct a columnar assembly. In contrast to DMP, CMP does not contain any strong inter-chain interactions. Hence, the slip planes (in the *ac* plane) run perpendicular to the herringbone layers, with apparent slip planes forming along the crystallographic *a* axis – *i.e.* the crystal needle axis.

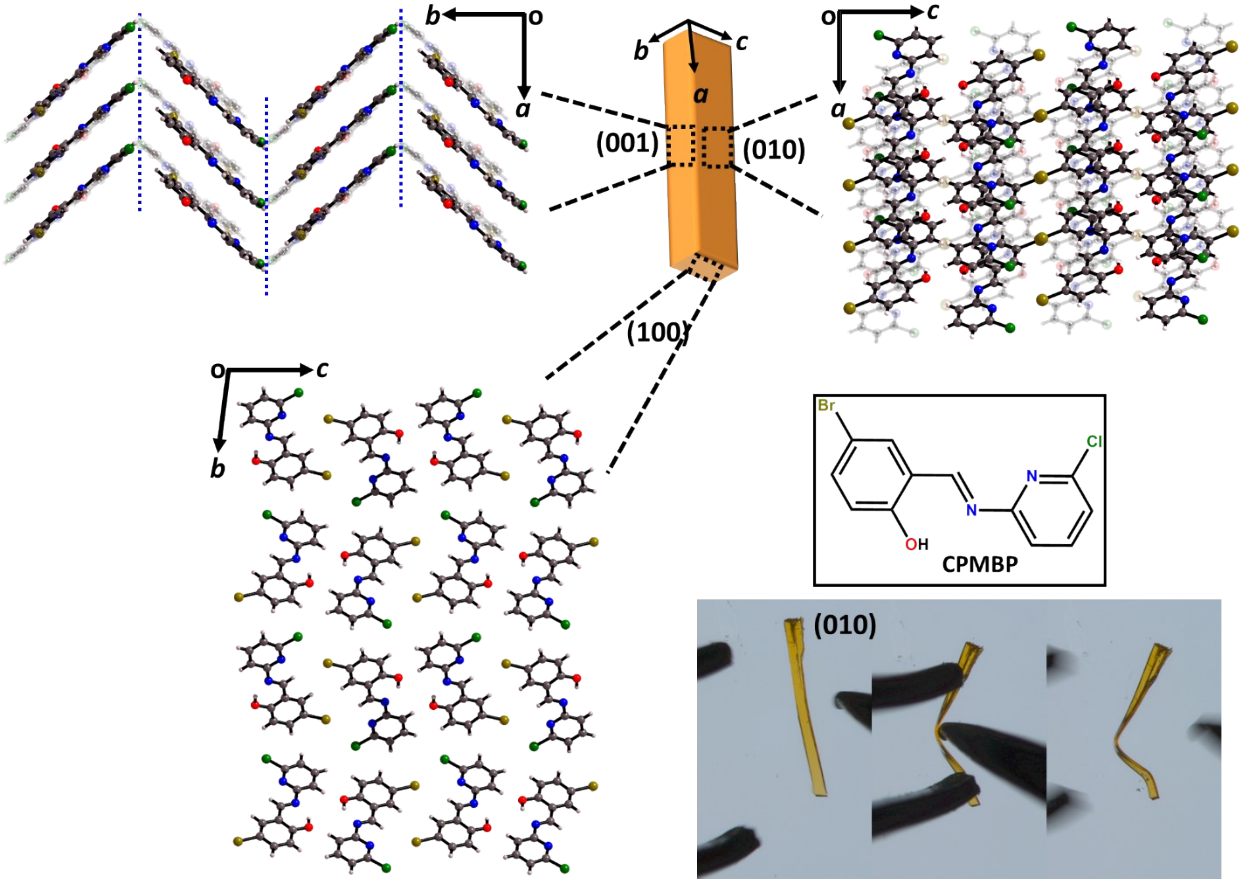

Fig. S10.1| Crystal packing for CMP, highlighting the structure at the (001), (010) and (100) faces. Details of crystal face indexing can be found in Reference [12]. The insets show the chemical structure of CMP and Fig.s of three point bending over the (010) crystal face.

When crystals of CMP were bent over the (010) face, exceptional and distinct plasticity was observed, **Fig. S10.1**. Plasticity over the (010) face corresponds to slip in parallel planes that run along the needle axis (the *ac* plane). Consistent with DMP, this corresponds to delamination of the planes perpendicular to the

herringboned layers. However, owing to the lack of strong interactions across these plans in CMP, plasticity is observed, rather than the elasto-plastic behaviour exhibited by DMP.

S10.2 Energy Framework Calculations of CMP| The intermolecular interactions in CMP were calculated using the energy framework calculation (**Fig. S10.2** and **Tables S10.2**). These calculations showed that the π···π stacking along the *a*-axis are the strongest interactions in CMP structure (-50.7 kJ.mol^{-1}), stabilizing the molecular columns that run along the needle axis. We note that the π ··· π interactions in CMP are *ca.* 2 kJ.mol^{-1} weaker than in the DMP structure (see **ESI S3.3**), suggesting that compression / expansion of the *a*-axis in CMP should occur more readily. In contrast, the total interaction energies along the herringbone chain (*b* axis) are -19.6 kJ.mol^{-1}, and the interchain interaction energies (along the *c* axis) are -73.5 kJ.mol^{-1}. Again we find that the interchain interaction energies in CMP are significantly lower than those in DMP (73.5 vs 82.3 kJ.mol^{-1}), indicating that delamination can occur much more readily in CMP than in DMP, presumably responsible for the markedly different mechanical behaviour of the materials.

Overall, the structure of CMP has anisotropic non-covalent interactions, consistent with qualitative models of plastic deformation[13] and the experimentally observed plastic deformation of CMP under three-point-bending (see Fig. S10.1 inset).

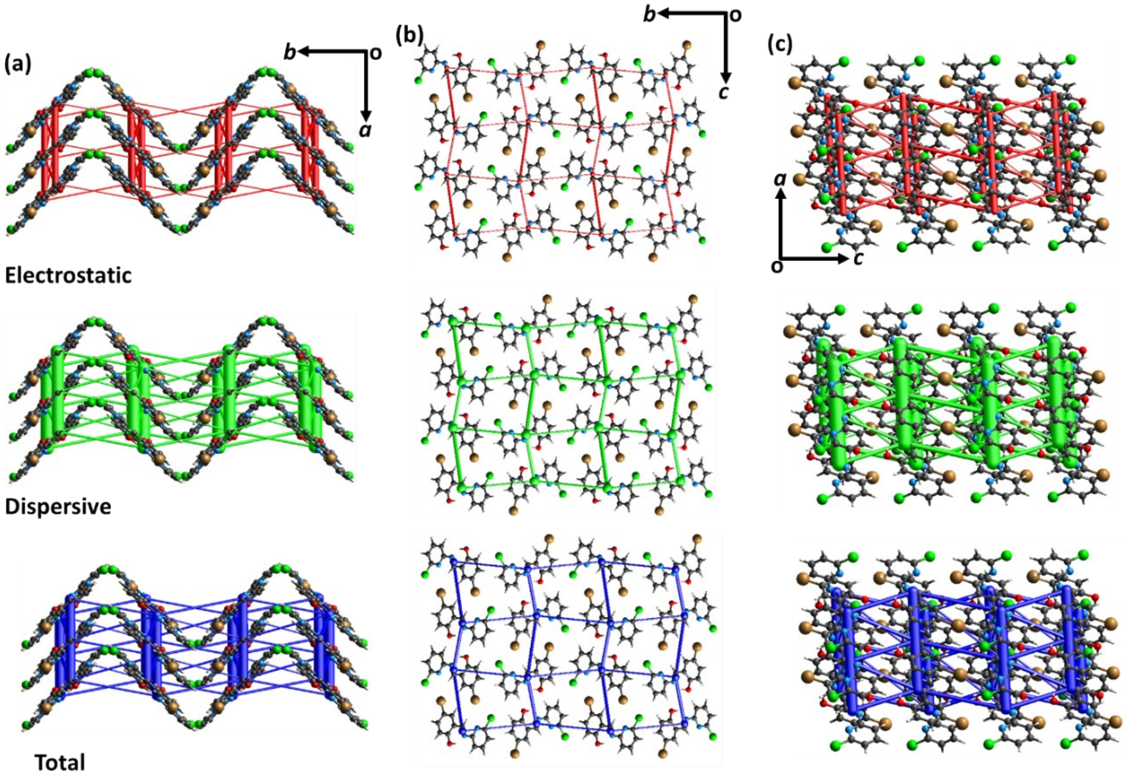

Fig. S10.2| Visualization of energy frameworks showing electrostatic (top, red), dispersion (middle, green) components and total interaction energy (below, blue) for CMP, in the (a) (001), (b) (100) and (c) (010) faces, respectively. The energy scale factor is 25 and the energy threshold is 5 kJ.mol^{-1}.

Table S10.2| Molecular structure pairs and the interaction energies (kJ.mol^{-1}) obtained from energy frameworks calculation for CMP. Scale factors are in the lower table.

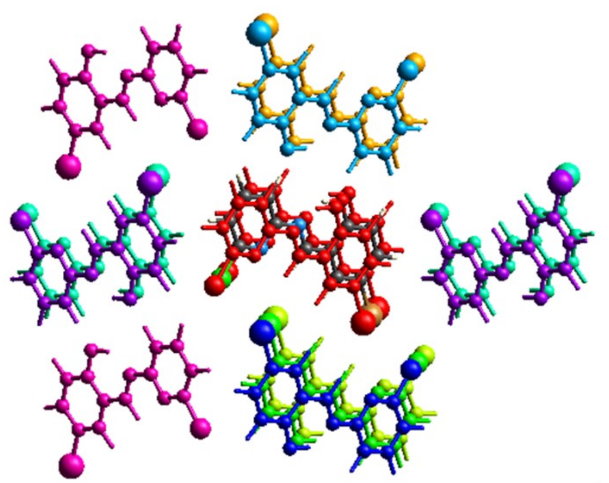

Total energies, only reported for two benchmarked energy models, are the sum of the four energy components, scaled appropriately (see the scale factor table below)

	N	Symop	R	Electron Density	E_ele	E_pol	E_dis	E_rep	E_tot
	2	x, y, z	4.44	B3LYP/DGDZVP	-30.9	-1.1	-77.8	81.8	-50.7
	1	-x, -y, -z	8.22	B3LYP/DGDZVP	-10.8	-3.6	-21.8	19.4	-21.0
	1	-x, -y, -z	8.01	B3LYP/DGDZVP	-4.9	-0.4	-12.2	9.2	-10.4
	1	-x, -y, -z	5.84	B3LYP/DGDZVP	-14.5	-1.9	-27.2	33.8	-19.5
	2	-x, y+1/2, -z+1/2	9.63	B3LYP/DGDZVP	-5.6	-0.8	-14.7	13.6	-10.9
	1	-x, -y, -z	9.04	B3LYP/DGDZVP	-10.2	-3.0	-17.4	13.6	-19.8
	1	-x, -y, -z	6.59	B3LYP/DGDZVP	-0.3	-0.3	-8.6	8.5	-2.8
	2	-x, y+1/2, -z+1/2	10.22	B3LYP/DGDZVP	-4.4	-0.6	-11.6	10.7	-8.7
	2	x, -y+1/2, z+1/2	11.38	B3LYP/DGDZVP	-1.9	-0.4	-4.1	3.9	-3.4

Scale factors for benchmarked energy models
See Mackenzie et al. IUCrJ (2017)

Energy Model	k_ele	k_pol	k_disp	k_rep
CE-HF ... HF/3-21G electron densities	1.019	0.651	0.901	0.811
CE-B3LYP ... B3LYP/6-31G(d,p) electron densities	1.057	0.740	0.871	0.618

S11 Anisotropic Compression of CMP

To better capture the effects of bending on the structure of CMP we conducted a series of *ab initio* simulations at the PBE-D2 level of theory, shown to reproduce the experimental crystal structure well, **Table S11.1**. Computational details are given in ESI S2. Our simulation methodology leads to an overall good agreement with the experimental unit cell geometry, slightly underestimating the experimental geometry, presumably due in large to the thermal expansion of the material.

Table S11.1| Optimization of CMP structure at PBE-D2 level of theory. Experimental data are taken from Ref [12], collected at 150 K.

Crystal Parameter	CMP
a (exp)	4.4398(3)
a (calc)	4.432689
Δa	-0.16%
b (exp)	19.0129(11)
b (calc)	18.530426
Δb	-2.54%
c (exp)	13.8958(13)
c (calc)	13.735123
Δc	-1.16%
β (exp)	92.180(3)
β (calc)	94.5583
$\Delta\beta$	2.57%
V(exp)	1172.14(13)
V (calc)	1124.641420
ΔV	-4.05%

To observe the effects of uniaxial compression on CMP we systematically expanded / contracted the unit cell along the crystallographic *a* axis, and allowed the remaining crystallographic parameters to relax, **Table S11.2**. The *a* axis was selected as it corresponds to the strained axis during bending (see **Fig. 4** in the main text). Upon compression, the herringboned layers clearly flatten, leading to elongation of the crystallographic *b* axis, and shortening of the crystallographic *c* axis, **Fig. S11.1**. In contrast, during expansion, the opposite trend is observed. This is consistent with the structural effects observed in DMP (see **Fig. 4** in the main text).

Table S11.2| Uniaxial compression of CMP structure at PBE-D2 level of theory.

$\Delta\beta$ /Å	ξ/%	a /Å	b /Å	c /Å	β	V	ΔEnergy /eV
+0.1	2.24467	4.532	18.207	13.738	94.097	1130.682	3.94809
+0.09	2.0202	4.523	18.235	13.738	94.131	1130.124	3.08449
+0.08	1.79574	4.513	18.268	13.737	94.178	1129.464	3.0001
+0.07	1.57127	4.503	18.299	13.737	94.214	1128.802	2.14526
+0.06	1.3468	4.493	18.331	13.738	94.259	1128.250	1.40429
+0.05	1.12233	4.482	18.361	13.738	94.306	1127.540	1.14819
+0.04	0.89787	4.472	18.394	13.737	94.352	1126.915	0.69453
+0.03	0.6734	4.462	18.430	13.736	94.400	1126.427	0.59981

+0.02	0.44893	4.452	18.460	13.736	94.445	1125.682	0.27603
+0.01	0.22447	4.442	18.490	13.736	94.493	1124.913	0.07651
0	0	4.433	18.530	13.735	94.551	1124.641	0
-0.01	-0.22447	4.424	18.571	13.734	94.633	1124.834	0.07669
-0.02	-0.44893	4.414	18.599	13.737	94.671	1123.960	0.27417
-0.03	-0.6734	4.404	18.629	13.739	94.704	1123.281	0.59146
-0.04	-0.89787	4.394	18.662	13.740	94.741	1122.729	0.68671
-0.05	-1.12233	4.384	18.696	13.740	94.783	1122.197	1.13704
-0.06	-1.3468	4.374	18.731	13.741	94.828	1121.693	1.72449
-0.07	-1.57127	4.364	18.762	13.740	94.883	1120.834	2.58554
-0.08	-1.79574	4.354	18.796	13.739	94.945	1120.100	2.54513
-0.09	-2.0202	4.344	18.834	13.738	94.998	1119.604	3.32491
-0.1	-2.22222	4.334	18.865	13.739	95.034	1118.894	3.57946
-0.2	-4.46689	4.234	19.188	13.736	95.486	1110.761	12.8127
-0.3	-6.71156	4.134	19.504	13.734	95.932	1101.373	27.86704

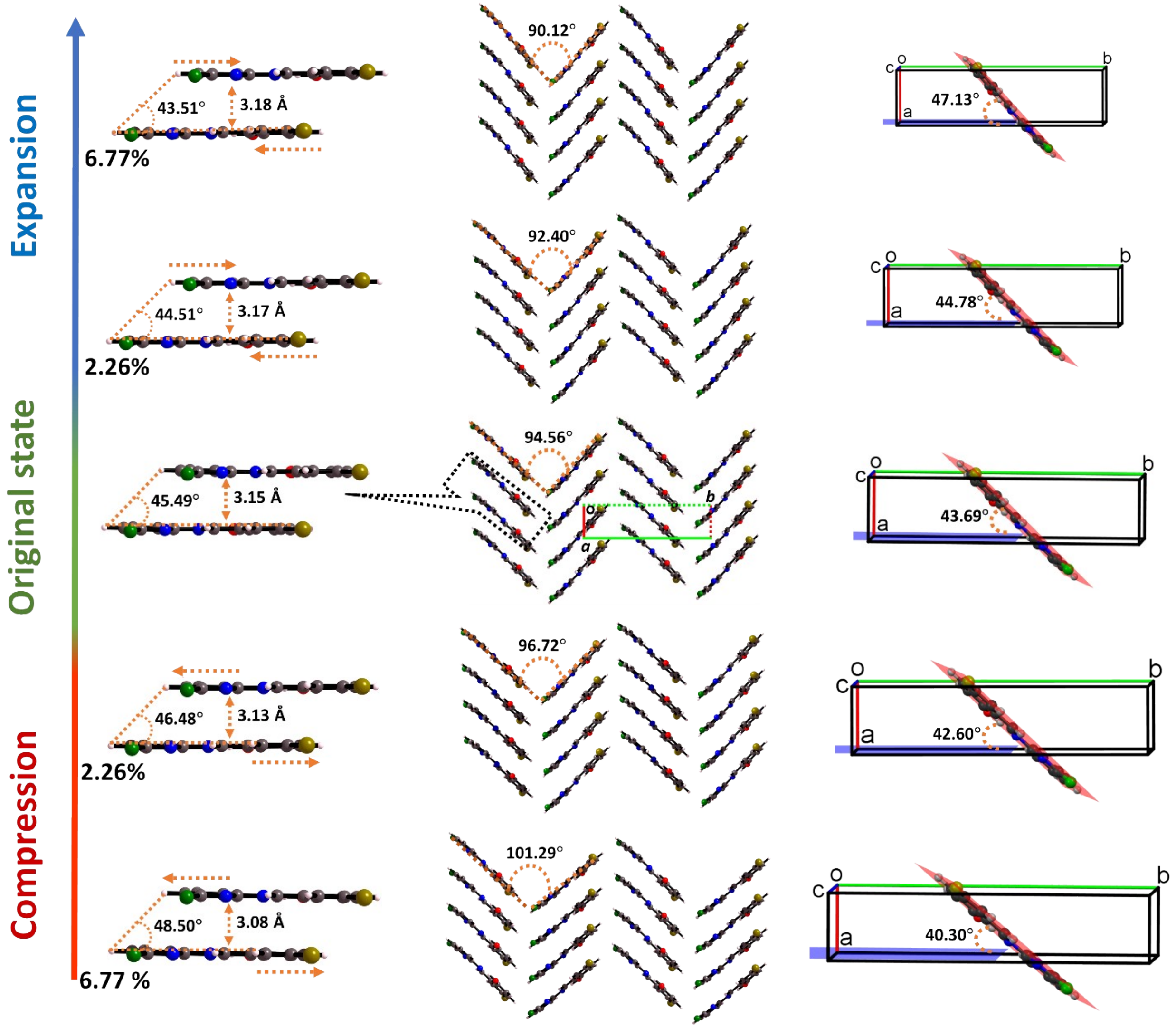

Fig. S11.3| Ab initio (PBE-D2) anisotropic compression and expansion of CMP along the crystallographic *a* axis. The shearing between adjacent layers of molecules, is shown, alongside the herringbone angle and the angle of molecules with respect to the (100) plane. We note excess extension and compression has been assumed to facilitate visualisation of structural effects.

The internal energy of CMP increases slowly with anisotropic compression, **Fig. S11.4**, at a slower rate than for DMP, **Fig. S9.1** and **Fig. 5** in the main text.

S12 SLIP PLANE ENERGIES IN DMP

The general model for bending suggests that the slip plane energy changes as a function of anisotropic compression along the bending axis. To explore this, we imposed systematic distortions of molecules along the respective slip planes by a factor $\zeta = \gamma/a$, where γ describes the Cartesian displacement in unit cell direction a (or *b, c*). A value of $\zeta = 1$ thus corresponds to a full translational unit of the unit cell dimension. In the perturbed geometry we performed single point energy calculations to obtain an estimated slip plane energy. At selected perturbations, the ionic positions and unit cell were left to relax. Relaxation was performed whilst freezing uniaxial compression and ionic positions in the slip direction. In this way, we ensure that structural relaxation occurs only in the dimensions that are not directly affected by the structural deformation of the model.

When considering the slip plane energies of DMP as a function of uniaxial compression/expansion (ξ), **Table S12.1**, it is clear that small amounts of distortion have marked impact on the subsequent ability of DMP to undergo plastic deformation. This is reflected in the Poisson's ratio of DMP (see **Section S13**), which may act as an indicator for the relative elasto-plastic threshold for flexible crystals. However, a larger family of crystals with this behaviour are first required.

Table S12.1| Effect of uniaxial compression/expansion (ξ) on the energy of the shear plane in DMP

		**Shear Energy /kJ.mol^{-1}**			
		ξ = -0.1	ξ =-0.4	ξ =0.4	ξ =0.1
Shear /%	0	0	0	0	0
	2	0.31109	0.57436	0.31381	0.34309
	4	1.24196	1.4098	1.20853	1.30012
	6	2.77257	2.78675	2.68179	2.87311
	8	4.8721	4.67984	4.74042	5.02958
	10	7.49628	7.05276	7.23607	7.72085

Structural analysis of the simulated structures suggests that the reduction in slip plane energy with anisotropic compression results from the elongation of the herringbone chains (*c* axis in DMP), as well as of the slip planes, Fig. S12.1.

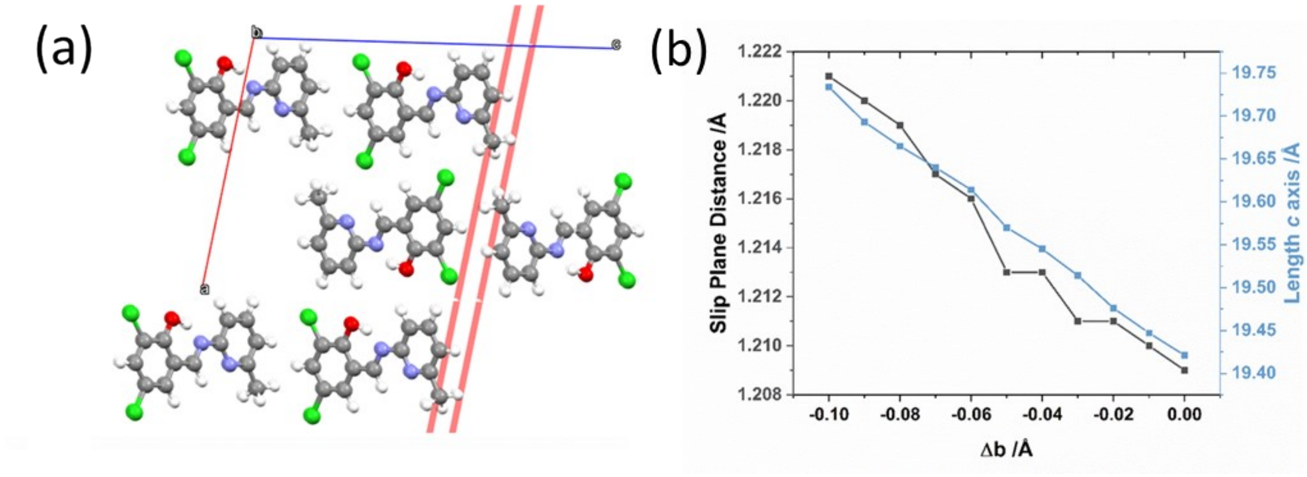

Fig. S12.1| Effect of uniaxial compression on the length of herringbone axis, and slip plane spacing. Slip planes are shown in the unit cell for (a) DMP) alongside structural data for (b) DMP.

S13 Simulated Elastic Tensors

S13.1 Elastic Tensors of DMP| Prior to simulating the elastic tensors, the geometry was relaxed at the corresponding level of theory, **Table S13.1**. The HF-3c method[9] reproduces very closely the low temperature unit cell of DMP, underestimating the volume by only 1.45%. Applying the scaling $s_8=0.7$, instead leads to a slight overestimation of the unit cell by only 1.96%. Optimisation with the PBE-D3(BJ) functional instead leads to a *ca.* 5% underestimation of the 100 K unit cell geometry, dominated by an underestimation of the unit cell *b*-axis (along the herringboned chains).

Table 13.1| Optimised unit cell geometries at the levels of theory used for elastic tensor simulation. Computational details are provided in **Section S2**.

	a	b	c	β	V
Exp 100 K	14.3805	4.2807	20.0178	101.319	1208.30
HF-3c	14.541	4.276	19.523	101.220	1190.774
ΔC(HF-3c) /%	+1.12	-0.11	-2.47	-0.10	-1.45
sHF-3c	14.659	4.345	19.729	101.326	1232.02
Δ(sHF-3c) /%	+1.94	+1.50	-1.46	+.01	+1.96
PBE-D3(BJ)	14.152	4.122	19.942	100.642	1143.193
Δ(PBE-D3(BJ)) /%	-1.59	-3.71	-0.38	-0.67	-5.39

Following from the relative unit cell volumes in **Table 13.1**, it is consistent to find that the elastic tensor (C_{ij}) and its inverse the stiffness tensor (S_{ij}) are softer for HF-3c as compared with sHF-c3, **Eqns S13.1-13.4** and **Table S13.2.** Benchmarking work by Spackman *et al.* suggests that minimal basis set parameterization of the Hartree-Fock method, HF-3c,[14] provide reasonable estimate of the elastic tensors for molecular materials. Consistent with the underestimated unit cell volume, PBE-D3(BJ) predicts a somewhat more rigid material as compared with both HF-3c approaches. We note our AFM measurements (**Fig. 2** in the main text) indicate a room temperature Young's modulus of *ca.* 6 GPa. This is generally consistent with our simulations when the effects of thermal expansion are considered (see for example **Table S13.1** the softening of elastic tensors when a change in volume of *ca* 4% is considered).

$$C_{ij}(HF-3c) = \begin{pmatrix} 21.523 & 3.882 & 6.478 & 0.000 & 4.769 & 0.000 \\ & 15.433 & 11.508 & 0.000 & -3.172 & 0.000 \\ & & 16.051 & 0.000 & 0.212 & 0.000 \\ & & & 8.166 & 0.000 & -2.041 \\ & & & & 6.886 & 0.000 \\ & & & & & 3.192 \end{pmatrix} \qquad \textbf{Eqn S13.1}$$

$$S_{ij}(HF-3c) = \begin{pmatrix} 0.0653 & -0.018 & -0.013 & 0.000 & -0.053 & 0.000 \\ & 0.184 & -0.126 & 0.000 & 0.101 & 0.000 \\ & & 0.159 & 0.000 & -0.054 & 0.000 \\ & & & 0.146 & 0.000 & 0.093 \\ & & & & 0.230 & 0.000 \\ & & & & & 0.373 \end{pmatrix} \qquad \textbf{Eqn S13.2}$$

$$C_{ij}(sHF-3c) = \begin{pmatrix} 18.390 & 3.023 & 5.337 & 0.000 & 4.317 & 0.000 \\ & 12.695 & 9.397 & 0.000 & -2.669 & 0.000 \\ & & 14.031 & 0.000 & 0.072 & 0.000 \\ & & & 6.933 & 0.000 & -1.749 \\ & & & & 5.856 & 0.000 \\ & & & & & 2.694 \end{pmatrix}$$ **Eqn S13.3**

$$S_{ij}(sHF-3c) = \begin{pmatrix} 0.078 & -0.022 & -0.015 & 0.000 & -0.067 & 0.000 \\ & 0.201 & -0.127 & 0.000 & 0.109 & 0.000 \\ & & 0.162 & 0.000 & -0.049 & 0.000 \\ & & & 0.172 & 0.000 & 0.112 \\ & & & & 0.271 & 0.000 \\ & & & & & 0.444 \end{pmatrix}$$ **Eqn S13.4**

Table S13.2| Elastic constants for DMP as obtained from *ab initio* simulations, showing the bulk modulus (*K*), shear modulus (*G*), and Young's modulus (*E*) according to the Voigt (V), Reuss (R) and Voigt-Reuss-Hill (H) schemes. All values are given in GPa.

	**HF-3c**	**sHF-3c**	**PBE-D3**
K_v	10.75	8.96	14.79
K_R	10.59	8.75	13.60
G_v	5.72	4.92	7.10
G_R	3.33	2.95	3.26
K_H	10.67	8.86	14.20
G_H	4.53	3.94	5.18
E_H	11.90	10.29	13.85
V_H	0.314	0.306	0.337

S13.2 Elastic Tensors of CMP| The geometry of CMP was relaxed at the corresponding level of theory, **Table S13.3**. The HF-3c method[9] underestimates the low temperature unit cell of CMP by 6.06%, providing marginally poorer agreement than for DMP. We note however, that the experimental geometry for CMP was measured at a higher temperature than for DMP, presumably accounting in part for this difference. By applying the scaling $s_8 = 0.7$, the sHF-3c method instead leads to a better agreement of the unit cell, underestimating it by 3.08%. The cell obtained using PBE-D3(BJ)/def2-mSVP is similar to that obtained by HF-3c, underestimating the 150 K unit cell by *ca.* 6%.

Table 13.3| Optimised unit cell geometries for CMP at the levels of theory used for elastic tensor simulation. Computational details are provided in **Section S2**.

	**a**	**b**	**c**	*β*	**V**
Exp 150 K	4.4399	19.0147	13.8964	92.184	1172.14
HF-3c	4.376	18.603	13.538	92.301	1101.105
Δ(HF-3c)	-1.439	-2.165	-2.579	+0.127	-6.060
sHF-3c	4.429	18.767	13.668	90.217	1136.010
Δ(sHF-3c)	-0.246	-1.303	-1.644	-2.134	-3.082
PBE-D3	4.393	18.455	13.625	94.352	1101.451
Δ(PBE-D3)	-1.056	-2.944	-1.953	+2.352	-6.031

$$C_{ij}(HF-3c)=\begin{pmatrix} 17.519 & 13.047 & 6.805 & 0.000 & 1.154 & 0.000 \\ & 16.943 & 7.814 & 0.000 & 0.408 & 0.000 \\ & & 26.300 & 0.000 & -1.154 & 0.000 \\ & & & 7.796 & 0.000 & -2.016 \\ & & & & 2.867 & 0.000 \\ & & & & & 8.497 \end{pmatrix}$$

Eqn S13.5

$$S_{ij}(HF-3c)=\begin{pmatrix} 0.140 & -0.103 & -0.007 & 0.000 & -0.045 & 0.000 \\ & 0.145 & -0.016 & 0.000 & 0.014 & 0.000 \\ & & 0.046 & 0.000 & 0.024 & 0.000 \\ & & & 0.137 & 0.000 & 0.032 \\ & & & & 0.374 & 0.000 \\ & & & & & 0.125 \end{pmatrix}$$

Eqn S13.6

$$C_{ij}(sHF-3c)=\begin{pmatrix} 14.436 & 11.511 & 5.312 & 0.000 & -0.108 & 0.000 \\ & 14.343 & 6.017 & 0.000 & 0.364 & 0.000 \\ & & 23.722 & 0.000 & -0.430 & 0.000 \\ & & & 6.319 & 0.000 & -2.107 \\ & & & & 1.274 & 0.000 \\ & & & & & 7.250 \end{pmatrix}$$

Eqn S13.7

$$S_{ij}(sHF-3c)=\begin{pmatrix} 0.228 & -0.188 & -0.002 & 0.000 & 0.118 & 0.000 \\ & 0.240 & -0.021 & 0.000 & -0.134 & 0.000 \\ & & 0.052 & 0.000 & 0.027 & 0.000 \\ & & & 0.176 & 0.000 & 0.051 \\ & & & & 1.085 & 0.000 \\ & & & & & 0.153 \end{pmatrix}$$

Eqn S13.8

$$C_{ij}(PBE-D3)=\begin{pmatrix} 16.850 & 14.516 & 7.980 & 0.000 & 2.591 & 0.000 \\ & 18.204 & 8.156 & 0.000 & 0.186 & 0.000 \\ & & 25.567 & 0.000 & -2.325 & 0.000 \\ & & & 5.774 & 0.000 & -1.351 \\ & & & & 4.301 & 0.000 \\ & & & & & 10.063 \end{pmatrix}$$

Eqn S13.9

$$S_{ij}(PBE-D3)=\begin{pmatrix} 0.286 & -0.208 & -0.039 & 0.000 & -0.184 & 0.000 \\ & 0.217 & 0.006 & 0.000 & 0.119 & 0.000 \\ & & 0.054 & 0.000 & 0.052 & 0.000 \\ & & & 0.178 & 0.000 & 0.024 \\ & & & & 0.367 & 0.000 \\ & & & & & 0.102 \end{pmatrix}$$

Eqn S13.10

Table S13.2| Elastic constants for CMP as obtained from *ab initio* simulations, showing the bulk modulus (*K*), shear modulus (*G*), and Young's modulus (*E*) according to the Voigt (V), Reuss (R) and Voigt-Reuss-Hill (H) schemes. All values are given in GPa.

	**HF-3c**	**sHF-3c**	**PBE-D3**
K_v	12.90	10.91	13.55
K_R	12.83	10.85	13.42
G_v	6.04	4.95	6.03
G_R	4.01	2.51	2.92
K_H	12.87	10.88	13.48
G_H	5.03	3.73	4.47
E_H	13.34	10.04	12.08
V_H	0.327	0.346	0.351

13.3 General Discussion of Mechanical Properties| The elastic tensor describes the deformability of the crystal structure and therefore likely holds significant information regarding the mechanical flexibility of single crystal materials. In our proposed model we consider the effects of uniaxial compression along the bending axis (*b* for DMP and *a* for CMP). Analysis of the Young's moduli, **Fig. S13.1**, along the respective directions shows that both materials respond similarly to uniaxial compression. It follows that the amount of stress accumulated by each material in response to bending-induced compression should be similar and thus does not account for the different mechanical behaviour of the material. The Poisson's ratio for each material do however suggest that the CMP unit cell should exhibit a more significant structural response from uniaxial compression. This is consistent with the finding that CMP slip planes are more affected by uniaxial compression than are those found in DMP.

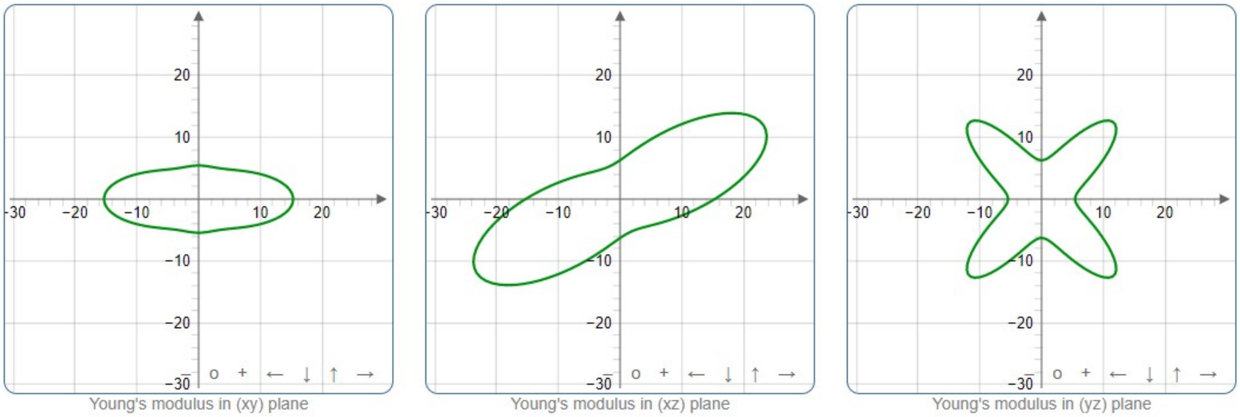

Fig. S13.1| 2D cuts of the Young's moduli for (top) DMP and (bottom) CMP. Values are shown in GPa. Data are shown as calculated at HF-3c level of theory and generated using ELATE.[15]

The most significant difference between the two flexible crystals is the magnitude of their shear moduli, **Fig. S13.2**. For the elasto-plastic crystal DMP, the shear modulus along the slip plane vector is *ca.* 2.7 GPa. In contrast, the modulus along the slip plane of CMP is only *ca.* 1 GPa. This strongly supports the relative ease of delamination in the latter, and its lack of elasto-plastic response.

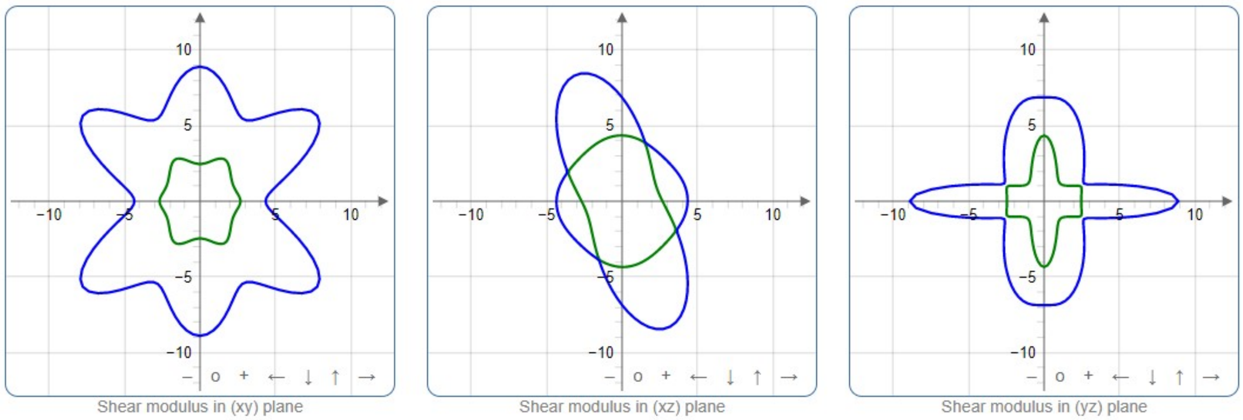

Fig. S13.2| 2D cuts of the shear moduli for (top) DMP and (bottom) CMP. Values are shown in GPa. Note that the surfaces represent the (blue) maximum and (green) minimum components in each direction. Data are shown as calculated at HF-3c level of theory and generated using ELATE.[15]

S15 References

1 T. Schmid and P. Dariz, *Heritage*, 2019, **2**, 1662–1683.

2 S. J. Clark, M. D. Segall, C. J. Pickard, P. J. Hasnip, M. I. J. Probert, K. Refson and M. C. Payne, *Z. Kristallogr. Cryst. Mater.*, , DOI:10.1524/zkri.220.5.567.65075.

3 H. J. Monkhorst and J. D. Pack, *Phys. Rev. B*, 1976, **13**, 5188–5192.

4 J. P. Perdew, K. Burke and M. Ernzerhof, *Phys. Rev. Lett.*, 1996, **77**, 3865–3868.

5 S. Grimme, *J. Comput. Chem.*, 2006, **27**, 1787–1799.

6 K. Refson, P. R. Tulip and S. J. Clark, *Phys. Rev. B*, 2006, **73**, 155114.

7 R. Dovesi, A. Erba, R. Orlando, C. M. Zicovich-Wilson, B. Civalleri, L. Maschio, M. Rérat, S. Casassa, J. Baima, S. Salustro and B. Kirtman, *WIREs Comput Mol Sci*, , DOI:10.1002/wcms.1360.

8 S. Grimme, J. Antony, S. Ehrlich and H. Krieg, *The Journal of Chemical Physics*, 2010, **132**, 154104.

9 R. Sure and S. Grimme, *J. Comput. Chem.*, 2013, **34**, 1672–1685.

10 M. J. Turner, S. P. Thomas, M. W. Shi, D. Jayatilaka and M. A. Spackman, *Chem. Commun.*, 2015, **51**, 3735–3738.

11 P. R. Spackman, M. J. Turner, J. J. McKinnon, S. K. Wolff, D. J. Grimwood, D. Jayatilaka and M. A. Spackman, *J Appl Crystallogr*, 2021, **54**, 1006–1011.

12 T. Feiler, B. Bhattacharya, A. A. L. Michalchuk, S.-Y. Rhim, V. Schröder, E. List-Kratochvil and F. Emmerling, *CrystEngComm*, 2021, **23**, 5815–5825.

13 G. R. Krishna, R. Devarapalli, G. Lal and C. M. Reddy, *J. Am. Chem. Soc.*, 2016, **138**, 13561–13567.

14 P. R. Spackman, A. Grosjean, S. P. Thomas, D. P. Karothu, P. Naumov and M. A. Spackman, *Angew Chem Int Ed*, , DOI:10.1002/anie.202110716.

15 R. Gaillac and P. Pullumbi, *J. Phys.*, 2016, 6.
